# Supplementary material for: Assessing emergence risk of double-resistant and triple-resistant genotypes of Plasmodium falciparum
Source: Nat Commun. 2024 Feb 15;15:1390. doi: 10.1038/s41467-024-45547-x (PMC10869733; doi:10.1038/s41467-024-45547-x)
Supplement: Supplementary file 1 — Supplementary Information [file 41467_2024_45547_MOESM1_ESM.pdf]

Assessing emergence risk of double-resistant and triple-resistant genotypes of *Plasmodium falciparum*

Supplementary Information

1 Specific MDR risks when MFT generates more risk than a cycling strategy

Figures 4 and 5 of the main text show that across the five maximally-resistant MDR genotypes defined in Table 1, MDR risk – as defined by the area under the genotype-frequency curve (AUC) – is lowest under MFT policies. However, this does not mean that MFT generates the lowest frequencies of each individual genotype, just the lowest sum of frequencies across all genotypes. Supplementary Figures 7 to 34 show the genotype frequencies in all epidemiological scenarios investigated here, and the underlined parts of each caption highlight the comparisons for which MFT is associated with higher MDR risk than one or both cycling policies.

In some scenarios, MDR generated slightly higher risk for the ASAQ double-resistant, the reason being that the simulations start with all genotypes carrying some resistance to AQ (as is true in reality). Therefore, with MFT using AQ for 33% of cases, 5-year cycling using AQ for 25% of cases, and adaptive cycling using AQ for about 10% to 15% of cases (depending on each individual simulation and when drug switches occur), MFT clearly puts the most selection pressure on AQ-resistant genotypes. Note that this is for a non-adaptive MFT approach that naively deploys three ACTs in equal amounts for 20 years without adjusting to changing levels of drug resistance. In Supplementary Table 1, we list all 8 of these scenarios and show that a simple change to a 50/50 MFT deployment of AL and DHA-PPQ results in substantial reductions in MFT's risk of driving an ASAQ double-resistant to high frequencies.

Note that the AUC numbers are not adjusted for prevalence. In other words, these numbers are in units of frequency-days not infection-days. This means that for some of the scenarios (e.g. 0.1% prevalence and 60% treatment coverage), an AUC value of 200 corresponds to 200 days of a genotype being fixed (frequency = 1.0), but this genotype could be fixed in a group of 50 parasite-positive individuals. For a scenario with MDR importation, some of these individuals may be recent imports meaning that their presence was not influenced by the drug policy in place.

Finally, a low absolute AUC value does correspond to low risk of an MDR genotype being generated during a 20-year period. AUC values in Supplementary Table 1 that are in the single digits correspond to an equivalent risk of a single-digit number of days (out of a twenty-year period) that a genotype is fixed in the population and thus guaranteed to be the one transmitted onward if a parasite-positive individual is bitten by a mosquito.

2 Definition of median simulation for Figures 6 and 7

To construct a mutation-flow diagram a representative simulation must be chosen out of the 100 simulated for each scenario. To do this, the median frequency of each genotype is calculated, by month, for the entire 20-year duration of the simulation. This gives $12 \times 20 \times 5 = 1200$ genotype-frequency data points for the five maximally-resistant genotypes. The simulation with the minimum absolute distance (summed over these 1200 data points) to the median frequencies of these five genotypes is labelled the median simulation.

Supplementary Table 1. Summary of cases when MFT generates more MDR risk for the ASAQ double resistant genotype (*pfkelch13* 580Y, *pfcrt* 76T, *pfmdr1* 86Y Y184).

$PfPR_{2-10}$	Treatment Coverage	Importation	Median number of MDR risk-days (AUC)	Median AUC from adaptive MFT approach (50/50 AL and DHA-PPQ deployment)
0.1%	20%	None	MFT: 4.30<br>5yr Cyc: 3.85<br>Ad Cyc: 2.28	Ad MFT: 1.22
0.1%	40%	None	MFT: 4.93<br>5yr Cyc: 3.70<br>Ad Cyc: 3.56	Ad MFT: 0.88
0.1%	60%	None	MFT: 0.23<br>5yr Cyc: 0.11<br>Ad Cyc: 0.07	Ad MFT: 0.06<br>(note that in this scenario malaria is eliminated after ten years)
1%	20%	None	MFT: 5.33<br>Ad Cyc: 3.84	Ad MFT: 2.95
1%	60%	None	MFT: 2.19<br>5yr Cyc: 2.15<br>Ad Cyc: 2.16	Ad MFT: 0.79
0.1%	20%	One MDR genotype per year	MFT: 6.74<br>5yr Cyc: 6.43<br>Ad Cyc: 5.15	Ad MFT: 2.11
0.1%	60%	One MDR genotype per year	MFT: 212.22<br>Ad Cyc: 182.79	MFT: 206.29<br>(note that this is a near-extinction scenario with tens of infections present, and importation playing an outsized role in determining genotype frequencies)
1%	20%	One MDR genotype per year	MFT: 7.00<br>5yr Cyc: 6.44<br>Ad Cyc: 4.71	Ad MFT: 3.67<br>(but MDR risk of DHA-PPQ double-resistant becomes worse)

Supplementary Table 2. Number of mutations to maximally-resistant MDR types defined in Table 1 of main text. The mutation counts below are from the 'median simulation' (see Section 2 for definition) of a $PfPR_{2\text{-}10}$ = 5% setting with 40% treatment coverage and no importation. In the 5-year cycling strategy, DHA-PPQ is used first, ASAQ second, AL third, and DHA-PPQ is deployed again in years 16-20.

		Years 0-5	Years 6-10	Years 11-15	Years 16-20
DHA-PPQ, AQ triple-resistant	MFT	19	38	123	318
	5-year cycling	51	75	83	372
DHA-PPQ, LUM triple-resistant	MFT	0	0	0	0
	5-year cycling	0	0	0	0
DHA-PPQ double-resistant	MFT	62	95	300	776
	5-year cycling	129	149	224	930
ASAQ double-resistant	MFT	935	670	897	951
	5-year cycling	888	680	841	891
AL double-resistant	MFT	0	0	0	0
	5-year cycling	0	0	0	0

3 Sensitivity of results to the emergence of a novel lumefantrine resistance locus

We re-evaluate certain scenarios under a hypothetical situation where a novel allele (at a novel locus) emerges conferring lumefantrine resistance, with this novel lumefantrine-resistant locus unlinked to any amodiaquine-resistant loci and with no effect on amodiaquine resistance or sensitivity. The two hypothetical alleles at this locus are called x (wild type) and X (lumefantrine-resistant), and we investigate three scenarios where the effect on lumefantrine resistance is modest, intermediate, or strong.

Modest effect. We start with the most-sensitive genotype to lumefantrine (76T, 86Y, Y184) and add an artemisinin-resistance mutation to drop the efficacy from the normal 93%–97% range. The efficacy of AL on genotype TYY--Y1x is 90.8% and we define a modest effect as one where the efficacy drops from 90.8% to 80.7% with a mutation from x to X at this new locus. The EC50 change required for this ~10% drop in efficacy between these two genotypes is an increase from 0.60 to 0.80. Thus, for all genotypes in the drug-by-genotype efficacy table, the lumefantrine EC50 was multiplied by 1.333 when mutating from x to X to generate a table of 64 new efficacies for AL when the allele X is present at the new locus. Under this modest effect of the new locus, the lowest AL efficacy, which occurs on the KNF--Y1X genotype, is 51.9%.

Intermediate effect. Efficacy of AL on genotype TYY--Y1x drops from 90.8% to 70.2% with a mutation from x to X. The EC50 change required for this ~20% drop in efficacy between these two genotypes is an increase from 0.60 to 0.95. For all other genotypes, EC50 was multiplied by 1.583 when mutating from x to X. In this scenario, the lowest AL efficacy, on KNF--Y1X, was 37.9%.

Strong effect. Efficacy of AL on genotype TYY--Y1x drops from 90.8% to 60.8% with a mutation from x to X. The EC50 change required for this ~30% drop in efficacy between these two genotypes is an increase from 0.60 to 1.07. For all other genotypes, EC50 was multiplied by 1.783 when mutating from x to X. In this scenario, the lowest AL efficacy, on KNF--Y1X, was 31.5%.

We repeated our analyses in six chosen scenarios (representative of the 15 prevalence-coverage combinations examined here). In almost all scenarios emergence of AL-resistant genotypes (double resistants) or AL+PPQ-resistant genotypes (triple resistants) did not occur in the 20 years the simulation was run, even with an additional lumefantrine resistance locus added. In one scenario (5% prevalence, 60% coverage), triple-resistants and double-resistants did emerge, however the AUC values for these resistants were low.

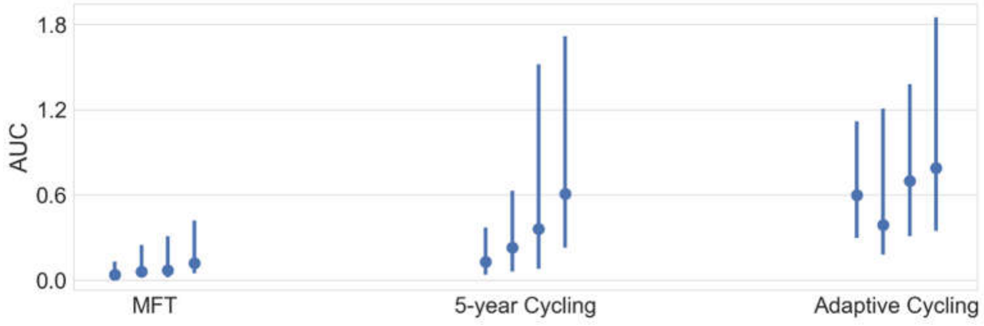

Supplementary Figure 1. AUC values for the double-resistant genotype to AL under three different treatment strategies, at 5% prevalence and 60% treatment coverage. AUC values here are for the double-resistant in Table 1 in the main text, across both x and X alleles. In each group, from left to right, the graphs show no effect, modest effect, intermediate effect, and strong effect of the novel lumefantrine locus. Circles show median values and lines are inter-quartile ranges from N=100 simulations.

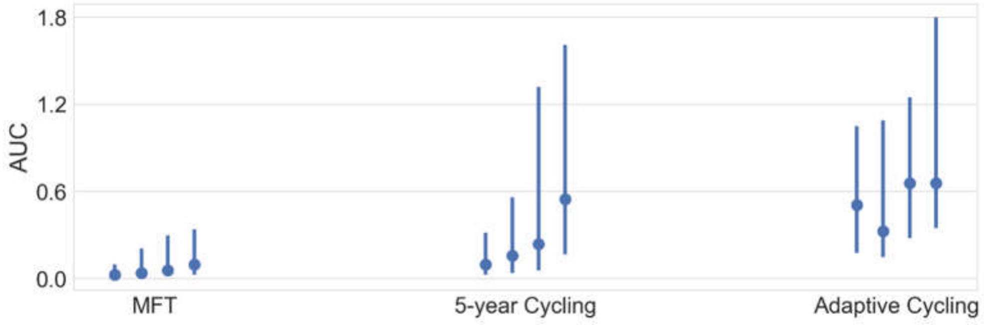

Supplementary Figure 2. AUC values for the triple-resistant genotypes to AL and PPQ under three different treatment strategies, at 5% prevalence and 60% treatment coverage. AUC values here are for the triple-resistant in Table 1 in the main text, across both x and X alleles. In each group, from left to right, the graphs show no effect, modest effect, intermediate effect, and strong effect of the novel lumefantrine locus. Circles show median values and lines are inter-quartile ranges from N=100 simulations.

4 Sensitivity of results to inferred drug efficacies

Results on the emergence of double-resistant and triple-resistant genotypes are likely to be sensitive to the reductions in efficacy conferred by double resistance and triple resistance. Some of these sensitivity analyses were done in a previous 3-team modeling exercise (Watson et al, Lancet Microbe, 3:701, 2022) that showed substantial differences in resistant genotypes' time to establishment when treatment efficacies were varied for these genotypes. Here, in order to assess the sensitivity to lumefantrine-resistance associated alleles specifically, we perform a sensitivity analysis (in the drug-by-genotype efficacy table, see section 4.1 of main text) on the effects of the Y184F allele and copy number variation in the *pfmdr1* gene.

We generated 3 additional drug-by-genotype efficacy tables (1) one where the Y184F locus was neutral with respect to lumefantrine resistance, (2) one where copy number variation (CNV) in *pfmdr1* has no effect on lumefantrine resistance, and (3) one where both Y184F and CNV of *pfmdr1* have no effect on lumefantrine resistance.

The results for emergence of AL double-resistants and AL-PPQ triple-resistants do not change from the original analysis in the paper, as the emergence of all AL-resistants in our model appears to be rare or slow. The median AUC values in rows 2 and 5 of Figures 2 and 3 in the main text are 0.00. These median values remain 0.00 under the three additional drug-by-genotype tables examined above.

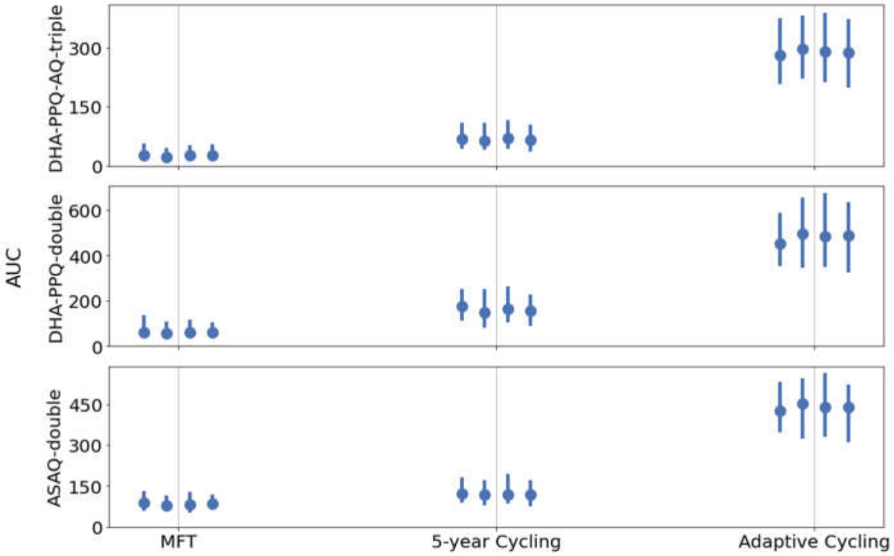

Supplementary Figure 3. AUC values for three resistant genotypes under three different treatment strategies, at 5% prevalence and 40% treatment coverage (as in Figure 2 of main text). In each group, from left to right, four different drug-by-genotype efficacy tables are used: (1) original table from main text, (2) table with no effect of Y184F on lumefantrine, (3) table with no effect of CNV of *pfmdr1* on lumefantrine, and (4) table with no effects of Y184F or CNV of *pfmdr1*. Circles show median values and lines are inter-quartile ranges from N=100 simulations.

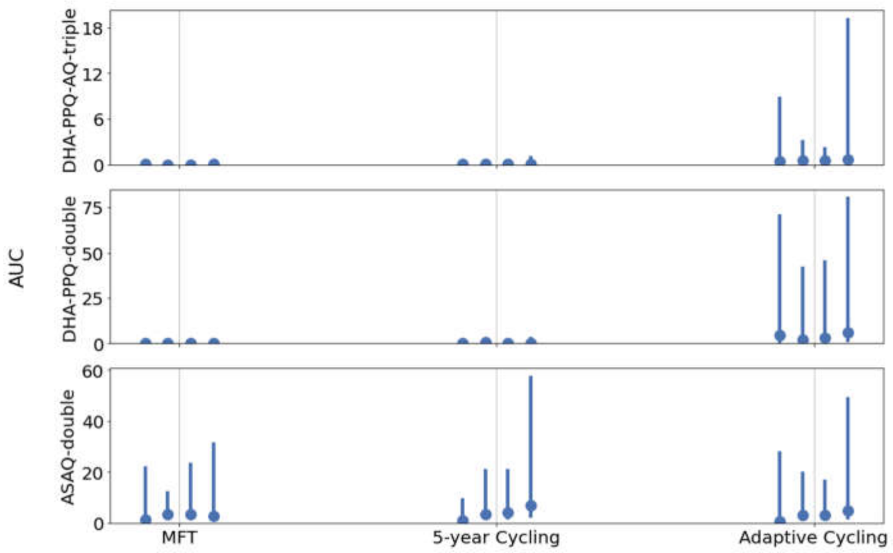

Supplementary Figure 4. AUC values for three resistant genotypes under three different treatment strategies, at 0.1% prevalence and 40% treatment coverage (as in Figure 3 of main text). In each group, from left to right, four different drug-by-genotype efficacy tables are used: (1) original table from main text, (2) table with no effect of Y184F on lumefantrine, (3) table with no effect of CNV of *pfmdr1* on lumefantrine, and (4) table with no effects of Y184F or CNV of *pfmdr1*. Circles show median values and lines are inter-quartile ranges from N=100 simulations.

5 General sensitivity analysis

In order to determine if any other evolutionary, ecological, or policy features have a strong influence on drug-resistance evolution, we conducted a general sensitivity analysis with Latin hypercube sampling on the following model parameters:

Supplementary Table 3. Parameters used for Latin hypercube sampling. Sampling ranges and distributions are shown. One thousand samples were drawn for a sensitivity analysis.

parameter	range	distribution
treatment coverage	0.20 – 0.90	uniform distribution for range
mutation rate	0.001983 – 0.01983	uniform distribution for range
cycling period	365 days – 1825 days	uniform distribution for range
mosquito biting rate (β)	0.0095 – 0.6100	uniform distribution on log(β)
annual fitness cost of resistance	11.9% – 28.9%	uniform distribution for range

to determine which parameters had the strongest influence on overall AUC across all five maximally-resistant genotypes from Table 1. This is the same AUC measure as is used in Figures 4 and 5 of the main text. Sensitivity analysis was done on fixed-period non-adaptive cycling strategies only. A total of 1000 parameter samples were chosen from the above ranges using a Latin hypercube sampling approach, and partial rank correlation coefficients (PRCC) between each parameter and the AUC resistance risk measure were calculated using the epiR package in R (https://cran.r-project.org/web/packages/epiR/index.html). The mosquito biting range was chosen to span the range of $PfPR_{2\text{-}10}$ from 0.1% to 50%.

In addition to the overall AUC risk measure across all five maximally-resistant genotypes, an additional PRCC plot was made (Supplementary Figure 6) to look at correlations between the above variables and the AUC of one of the triple-resistants.

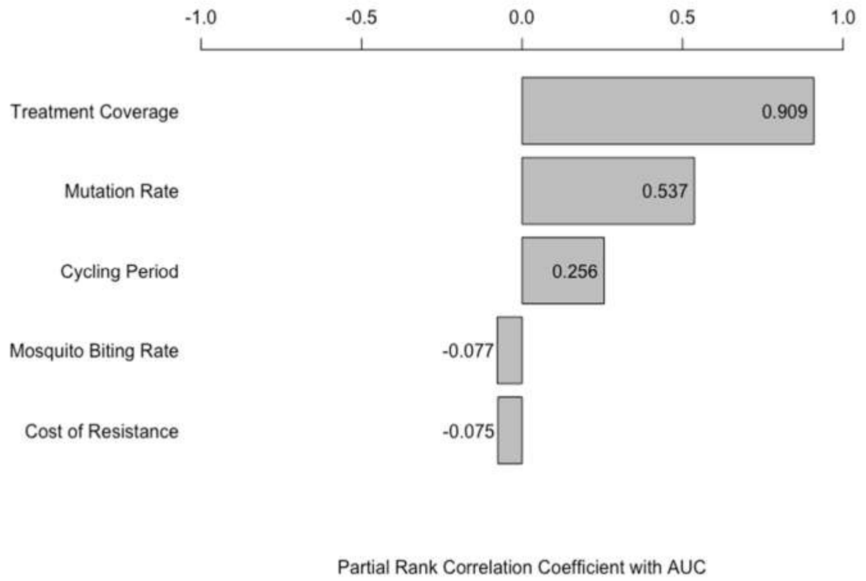

Supplementary Figure 5. Partial rank correlation coefficients between five parameters of interest and the AUC measure of total double- and triple-resistance risk. All five correlation coefficient are statistically non-zero ($p < 0.02$). As expected, treatment coverage has the strongest association with resistance risk, and higher mutation rate is also associated with increased frequency of double-resistance and triple-resistance. Cycling period is positively associated with increased multi-drug resistance risk showing that long cycling periods – despite creating a constant evolutionary environment for one or two types of resistance – are nevertheless associated with higher MDR risk.

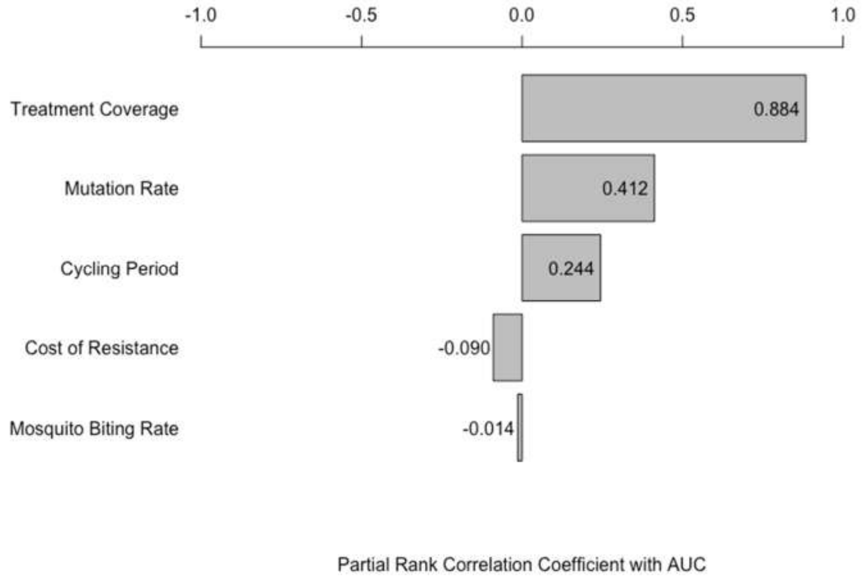

Supplementary Figure 6. Partial rank correlation coefficients between five parameters of interest and the AUC measure for the DHA-PPQ-AQ triple-resistant only. The first four coefficients top-to-bottom are statistically non-zero ($p < 0.005$). Cycling period is positively associated with increased triple-resistance risk showing that long cycling periods – despite never generating simultaneous triple pressure on three different resistance phenotypes – are nevertheless associated with higher triple-resistance risk.

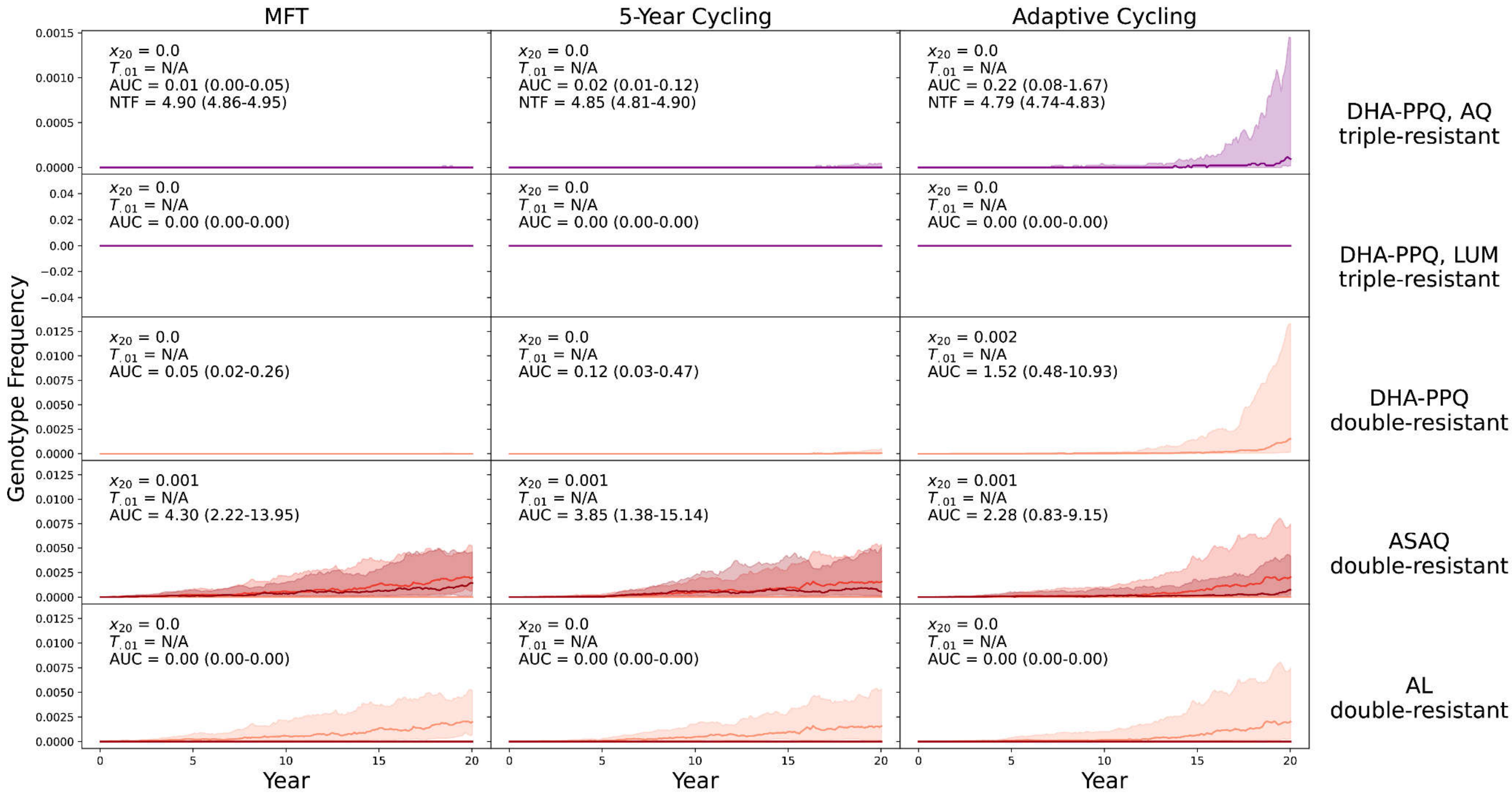

Supplementary Figure 7. PfPR$_{2\text{-}10}$ = 0.1% and treatment coverage = 20%. No importation. Each row shows the median trajectories, with shaded areas showing interquartile ranges, of the double-resistant or triple-resistant labelled at the right. In the bottom two rows, dark red corresponds to quadruple-mutant double-resistance, medium red corresponds to triple-mutant double-resistance, and light red corresponds to double-mutant double-resistance. The columns correspond to the three different drug deployment strategies. The final mutant genotype frequency after 20 years (x_{20}), the time until this genotype reaches 0.01 frequency ($T_{.01}$), the number of MDR risk-days for this mutant (AUC), and the number of treatment failures (NTF) per 100 persons per year are shown in each panel. Note that for the ASAQ double-resistant, MFT has a higher AUC value than either cycling strategy.

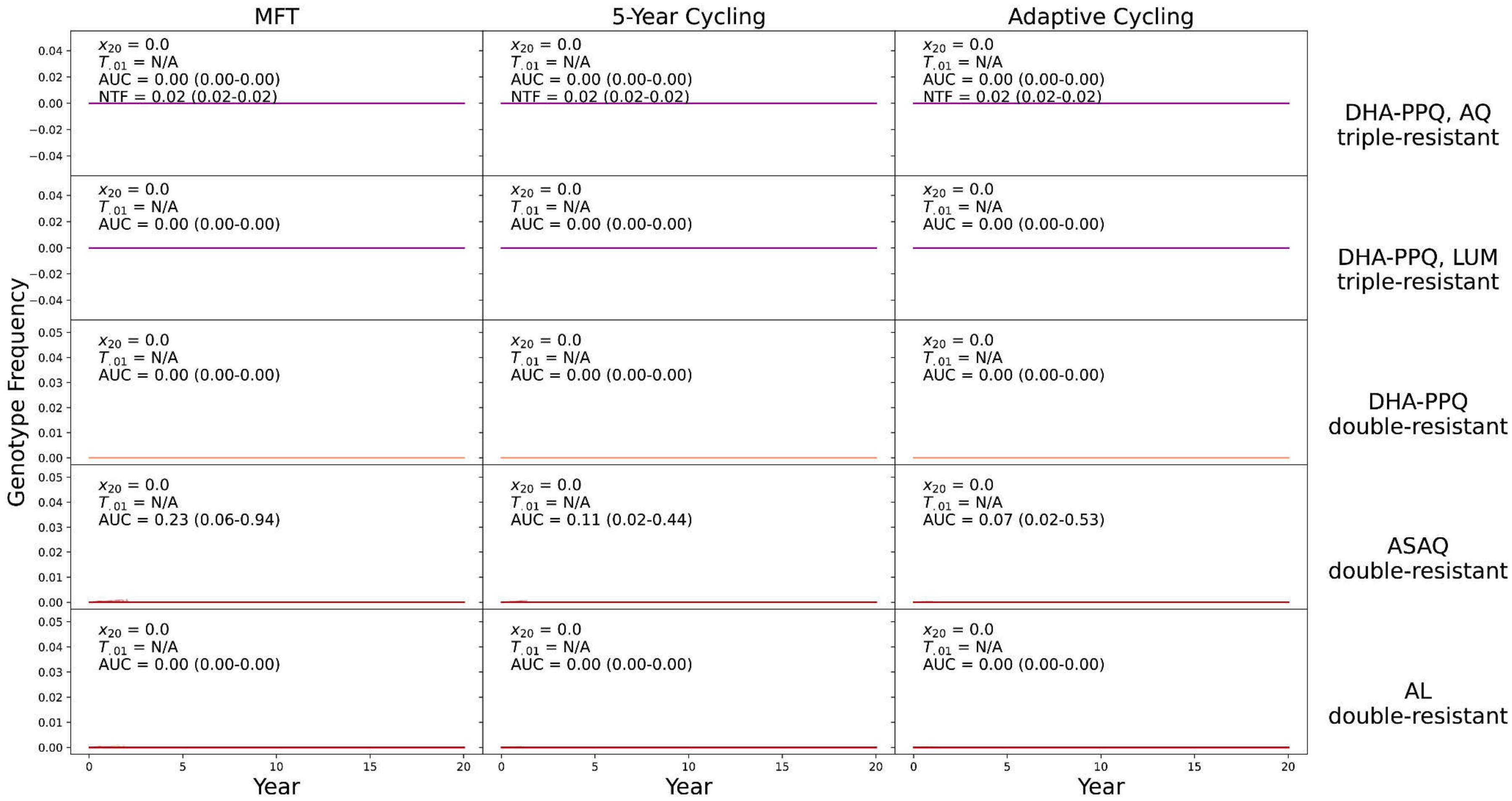

Supplementary Figure 8. PfPR$_{2\text{-}10}$ = 0.1% and treatment coverage = 60%. No importation. 40% coverage is shown in Figure 3 of the main text. Note that for the ASAQ double-resistant, MFT has a higher AUC value than either cycling strategy. In these runs, malaria is effectively eliminated in more than 95% of simulations, for all strategies. The AUC values show the sums, in units of frequency-days, during years 0 to 10, prior to elimination.

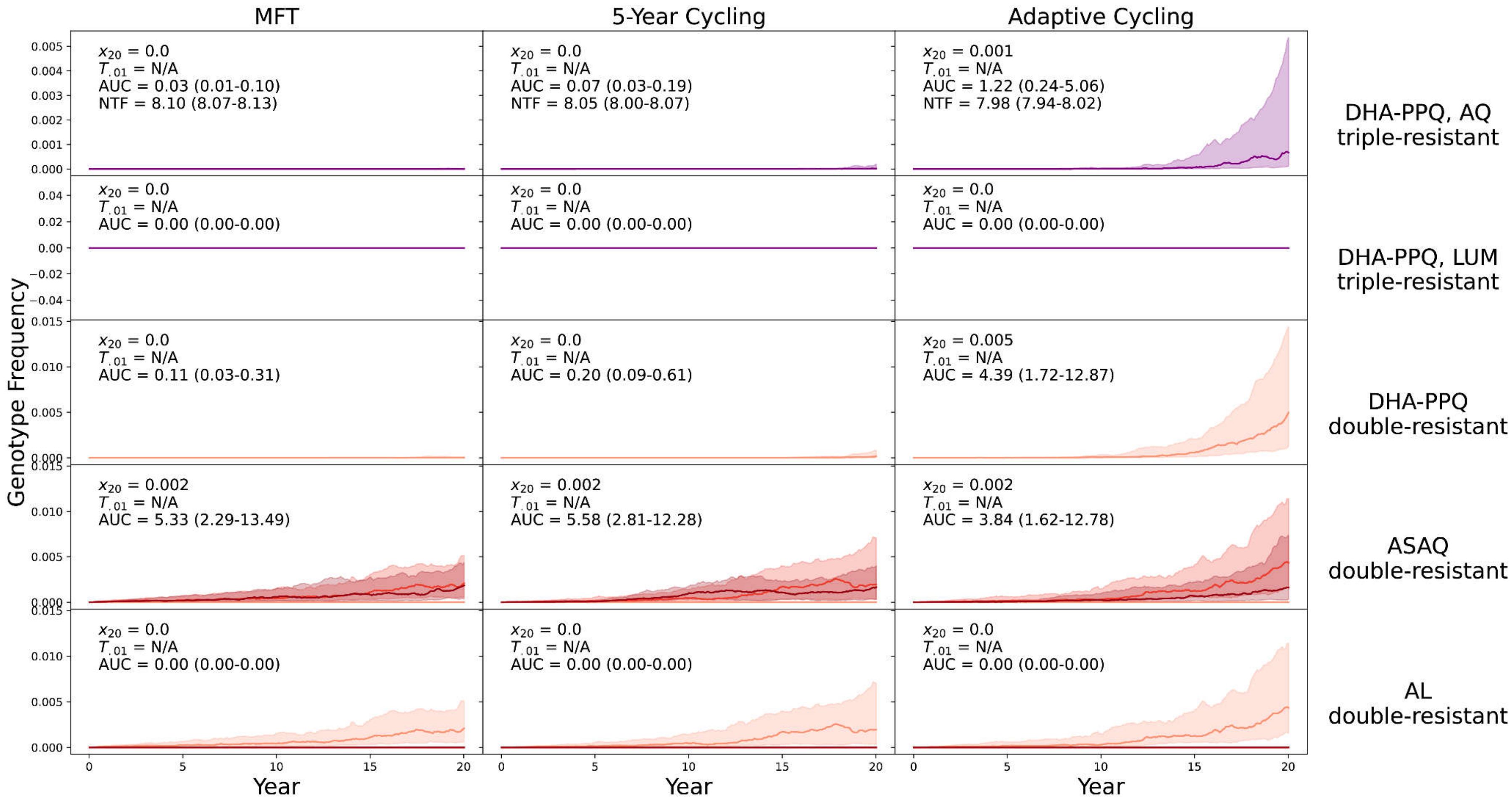

Supplementary Figure 9. PfPR$_{2\text{-}10}$ = 1% and treatment coverage = 20%. No importation. Note that for the ASAQ double-resistant, MFT has a higher AUC value than the adaptive cycling strategy.

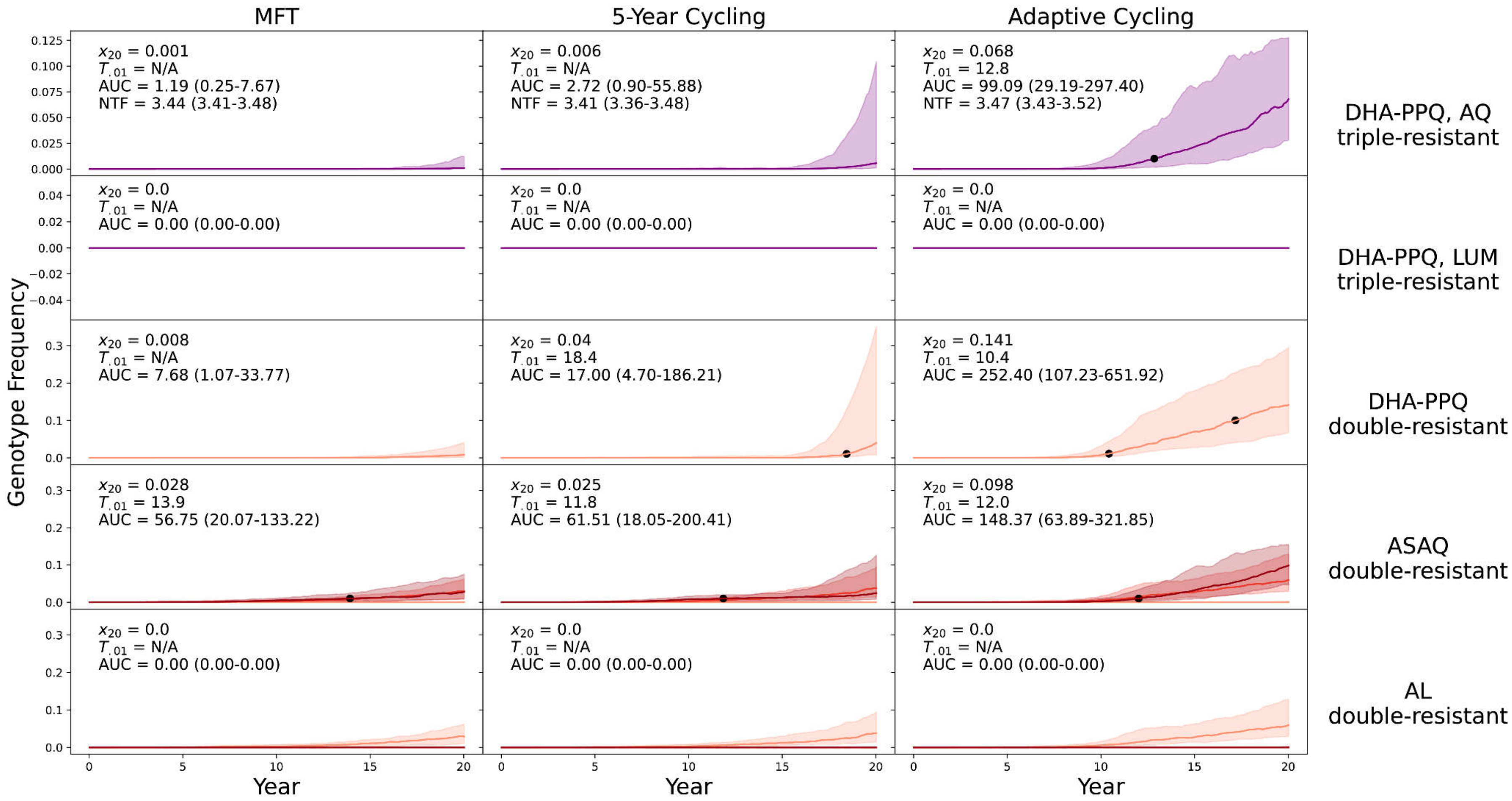

Supplementary Figure 10. PfPR$_{2\text{-}10}$ = 1% and treatment coverage = 40%. No importation.

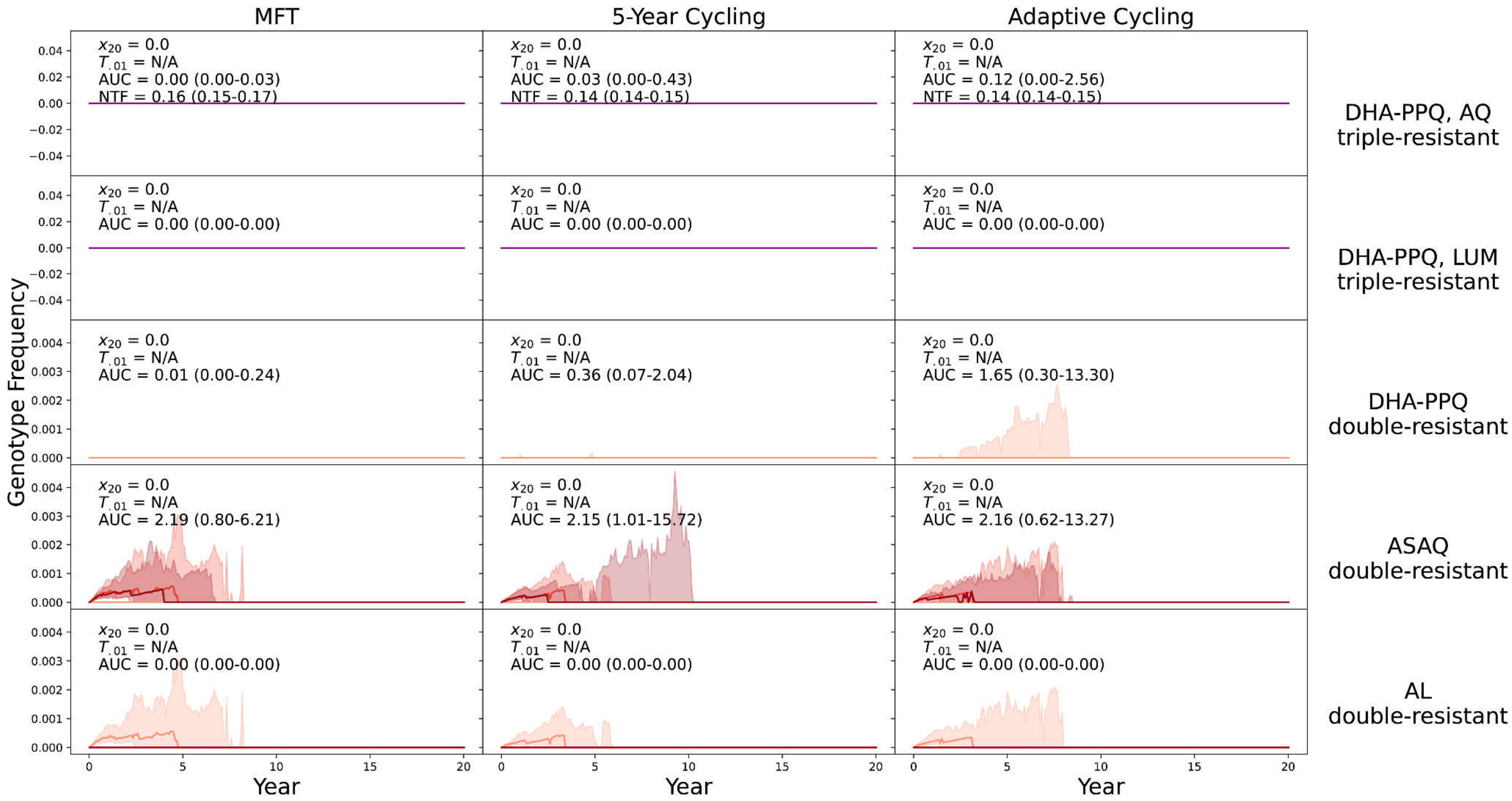

Supplementary Figure 11. PfPR$_{2\text{-}10}$ = 1% and treatment coverage = 60%. No importation. <u>Note that for the ASAQ double-resistant, MFT has a higher AUC value than either cycling strategy</u>.

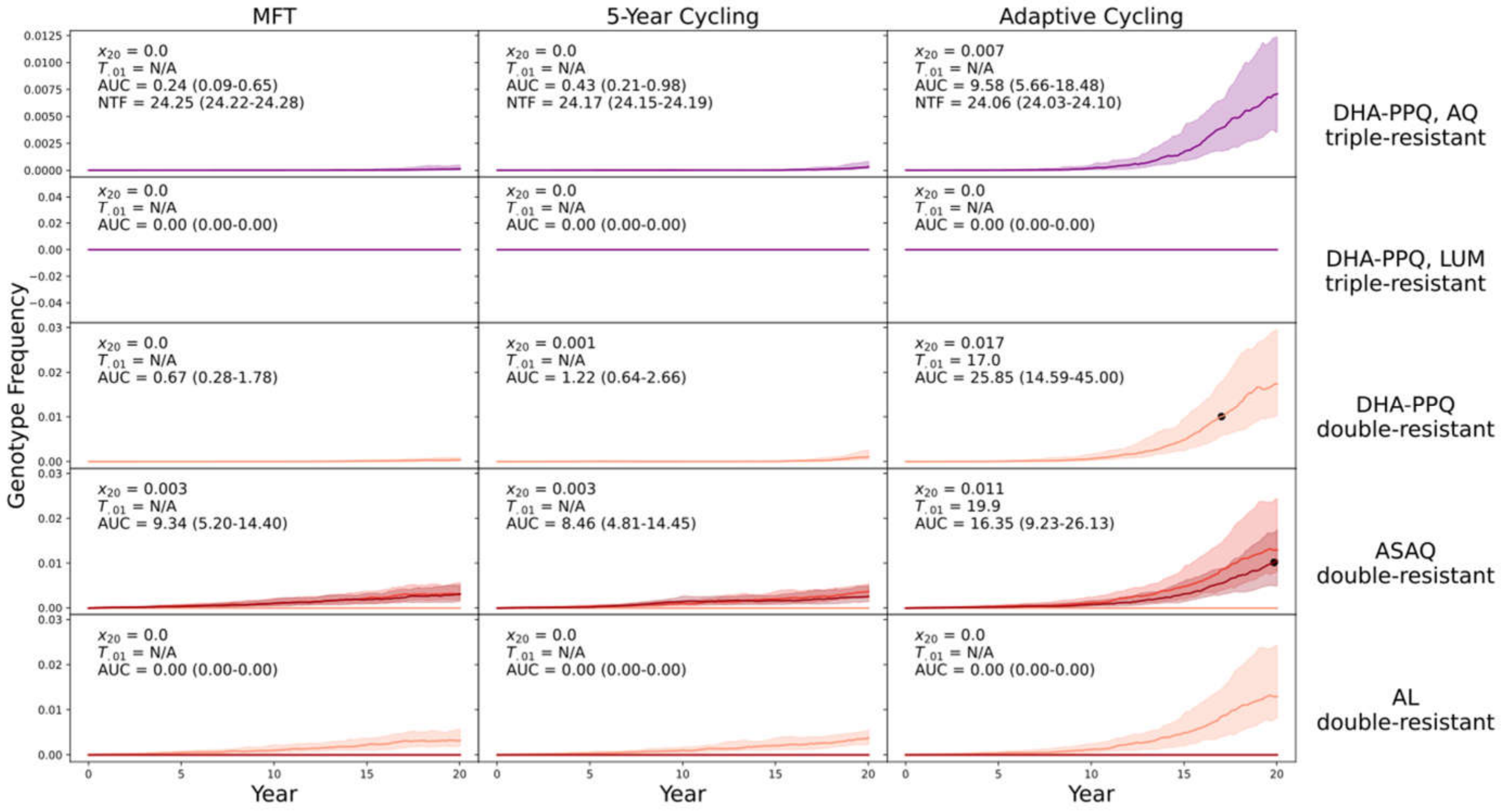

Supplementary Figure 12. PfPR$_{2\text{-}10}$ = 5% and treatment coverage = 20%. No importation.

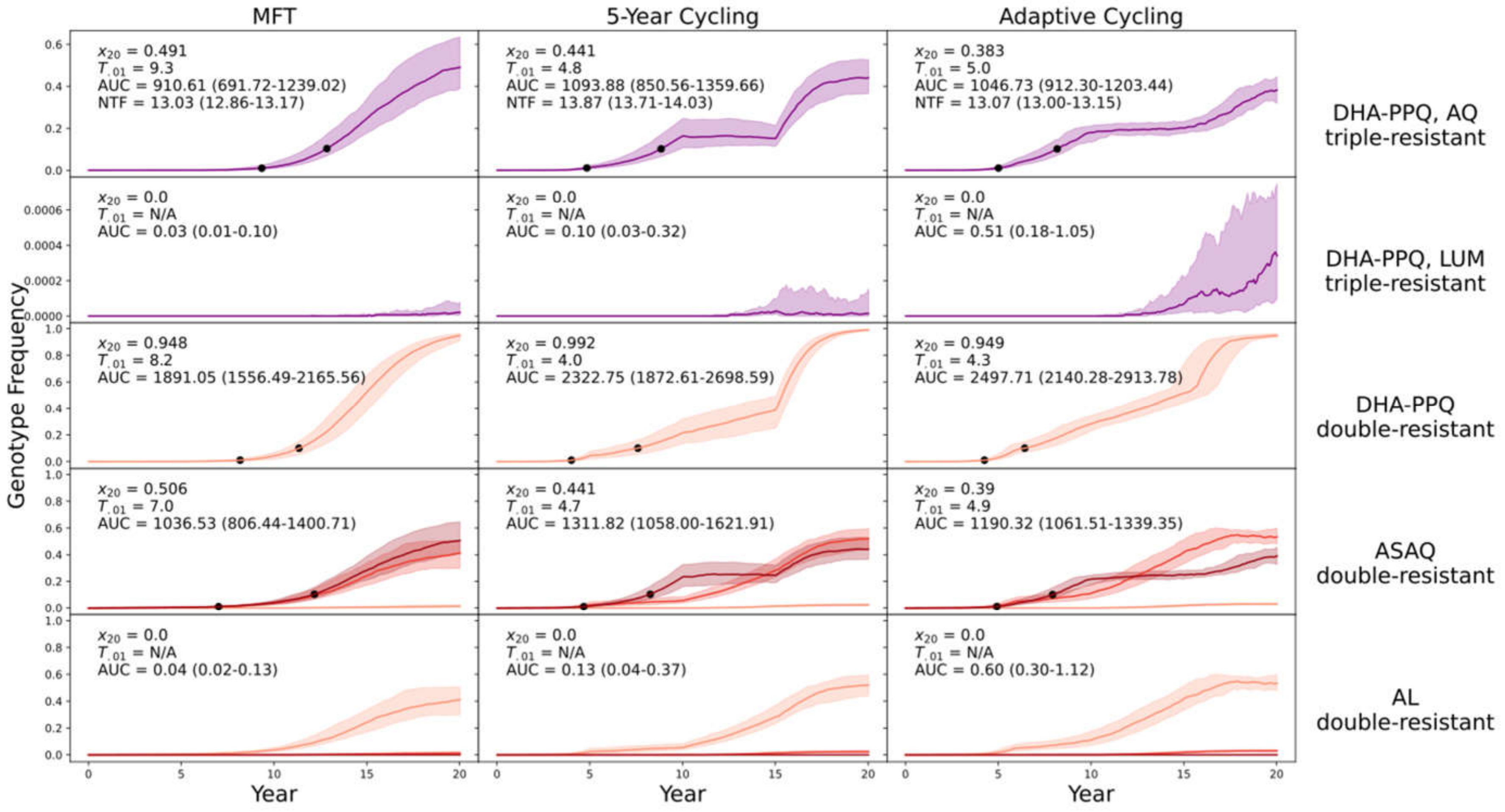

Supplementary Figure 13. PfPR$_{2\text{-}10}$ = 5% and treatment coverage = 60%. No importation. 40% coverage shown in Figure 2 of main text.

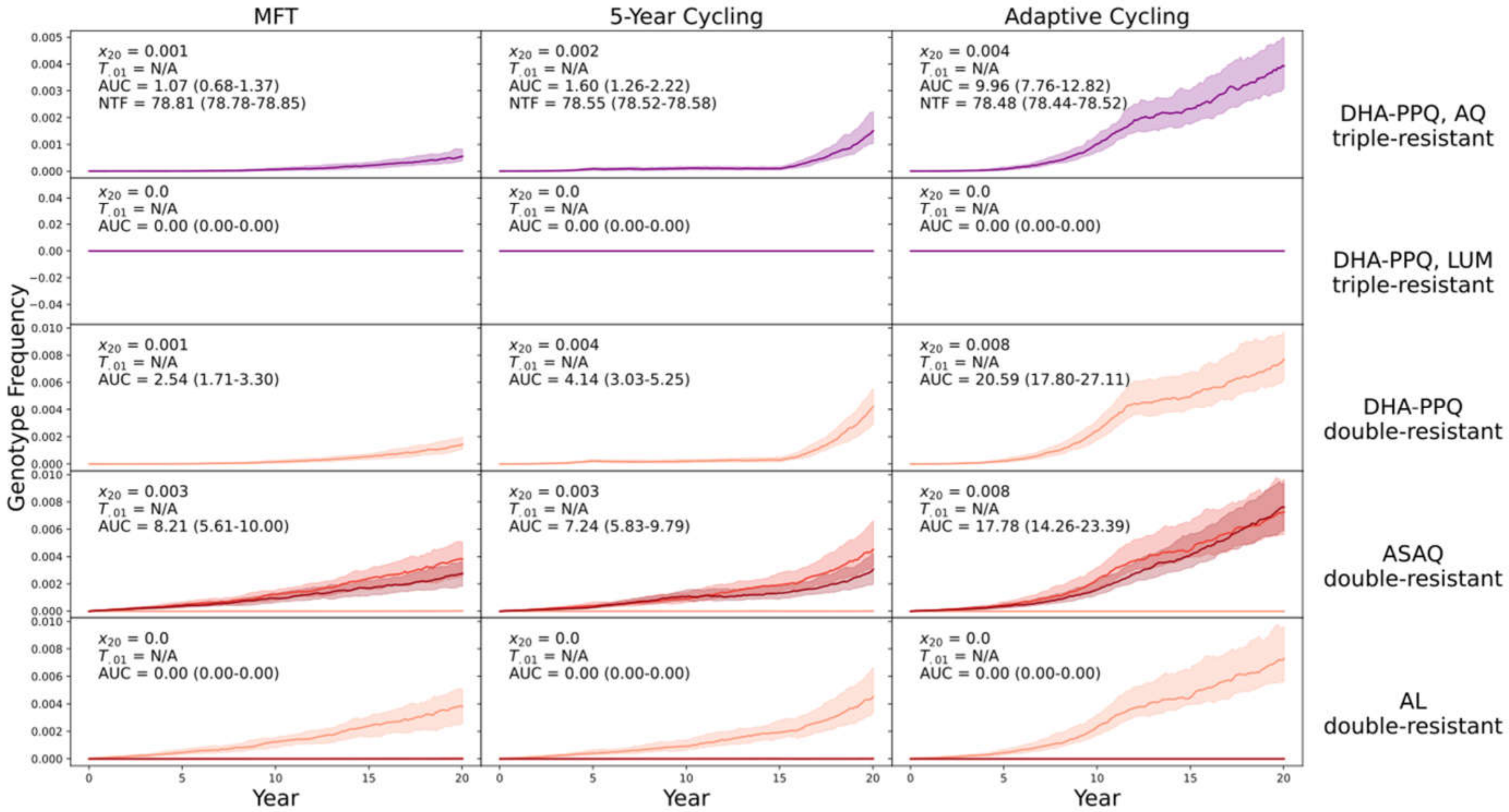

Supplementary Figure 14. PfPR$_{2\text{-}10}$ = 25% and treatment coverage = 20%. No importation.

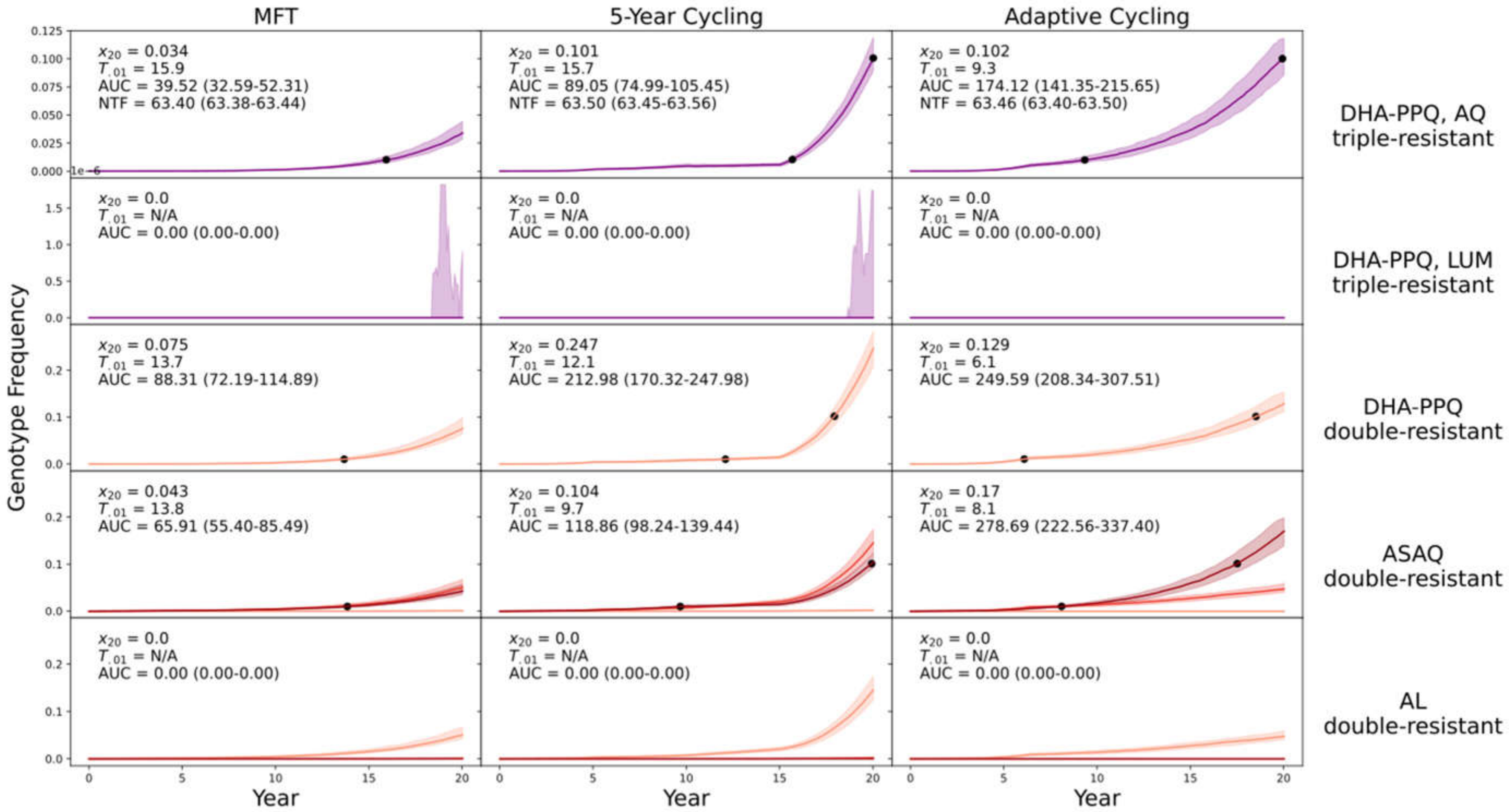

Supplementary Figure 15. PfPR$_{2-10}$ = 25% and treatment coverage = 40%. No importation.

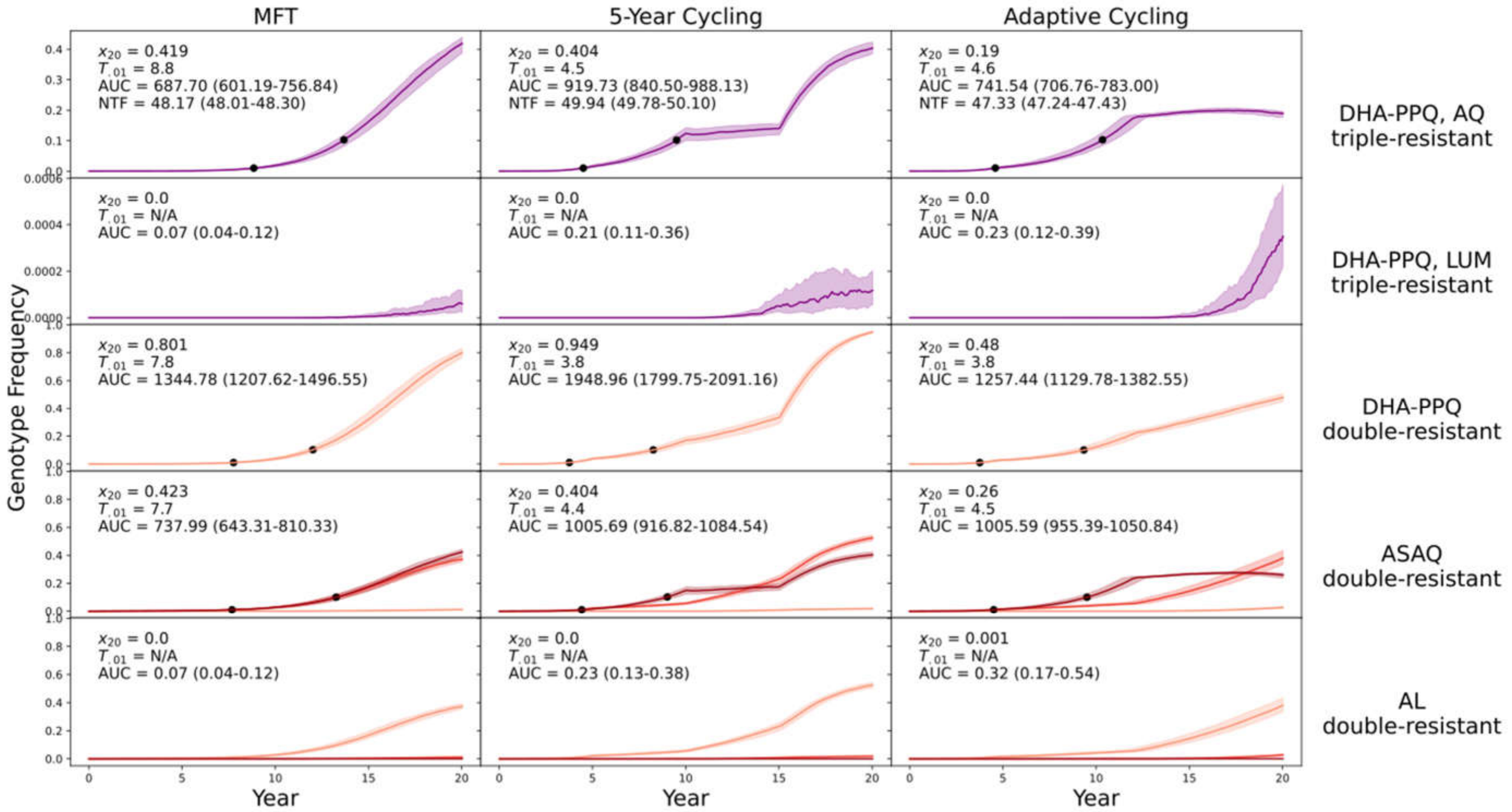

Supplementary Figure 16. $PfPR_{2\text{-}10}$ = 25% and treatment coverage = 60%. No importation. Note that for the DHA-PPQ double-resistant, MFT has a higher AUC value than the adaptive cycling strategy.

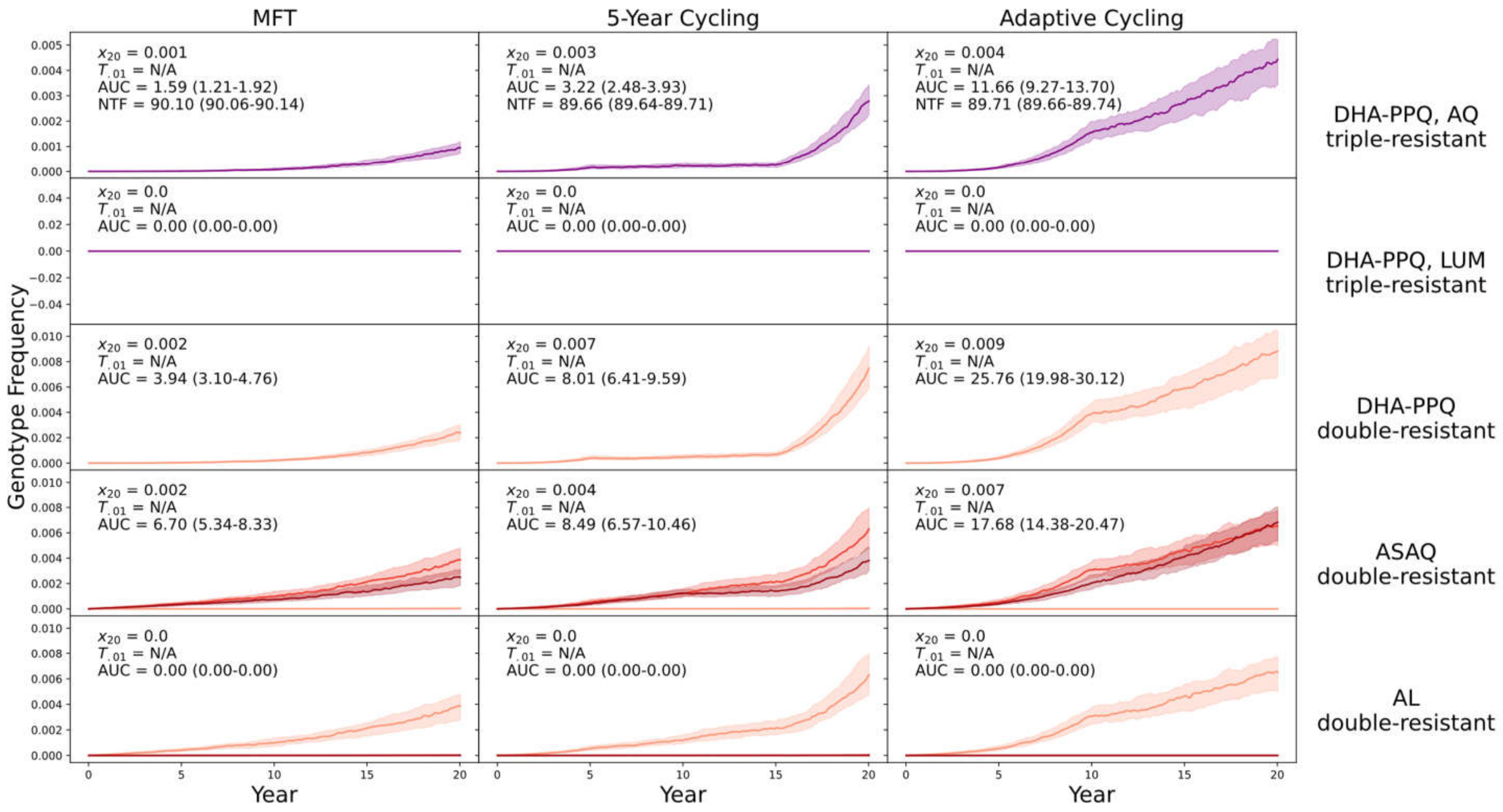

Supplementary Figure 17. PfPR$_{2\text{-}10}$ = 50% and treatment coverage = 20%. No importation.

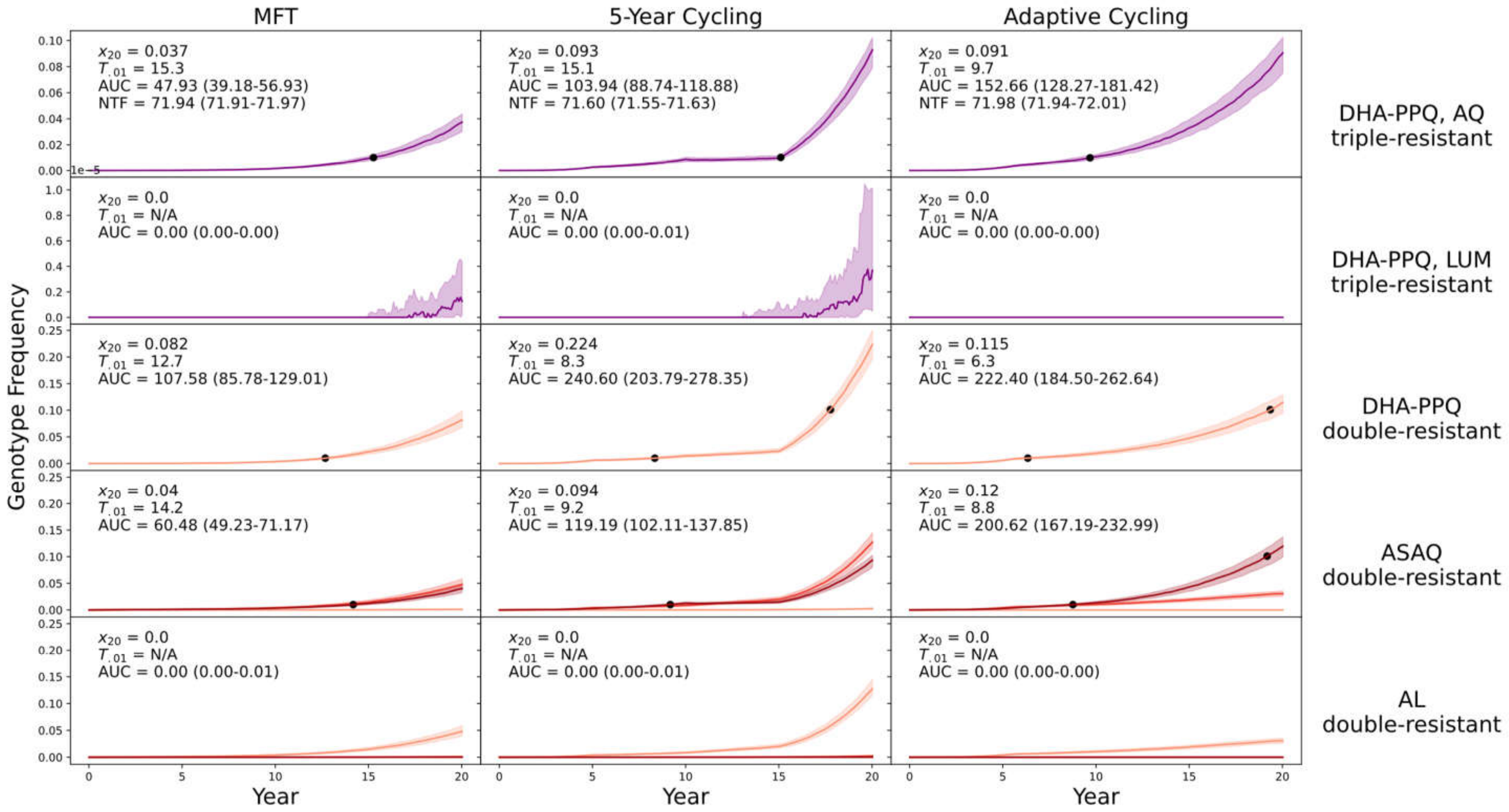

Supplementary Figure 18. PfPR$_{2\text{-}10}$ = 50% and treatment coverage = 40%. No importation.

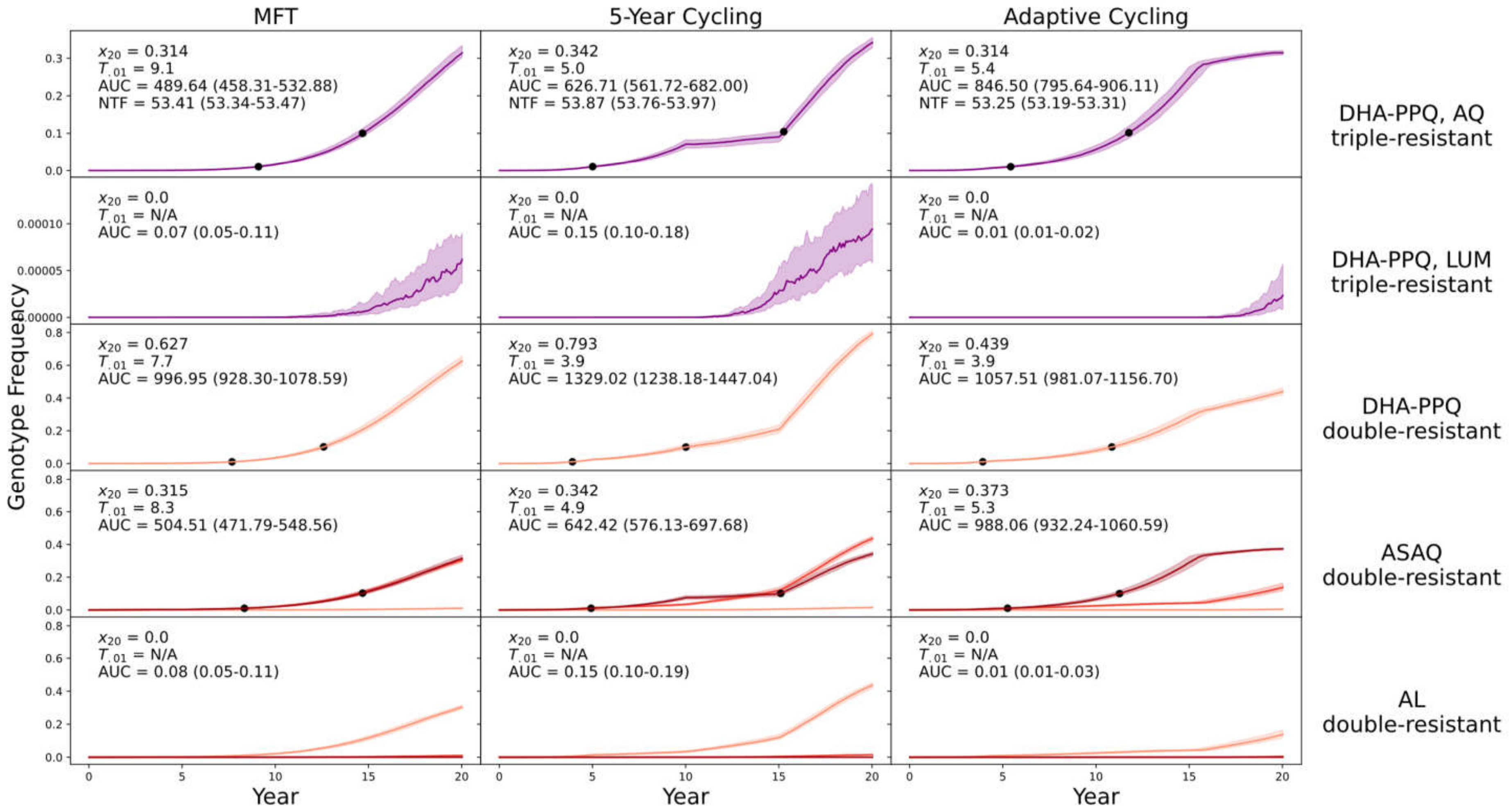

Supplementary Figure 19. PfPR$_{2\text{-}10}$ = 50% and treatment coverage = 60%. No importation. Note that for the AL double-resistant and the DHA-PPQ-LUM triple-resistant, MFT has a higher AUC value than the adaptive cycling strategy.

Fifteen epidemiological scenarios with importation (summarized in Figure 5 of main text)

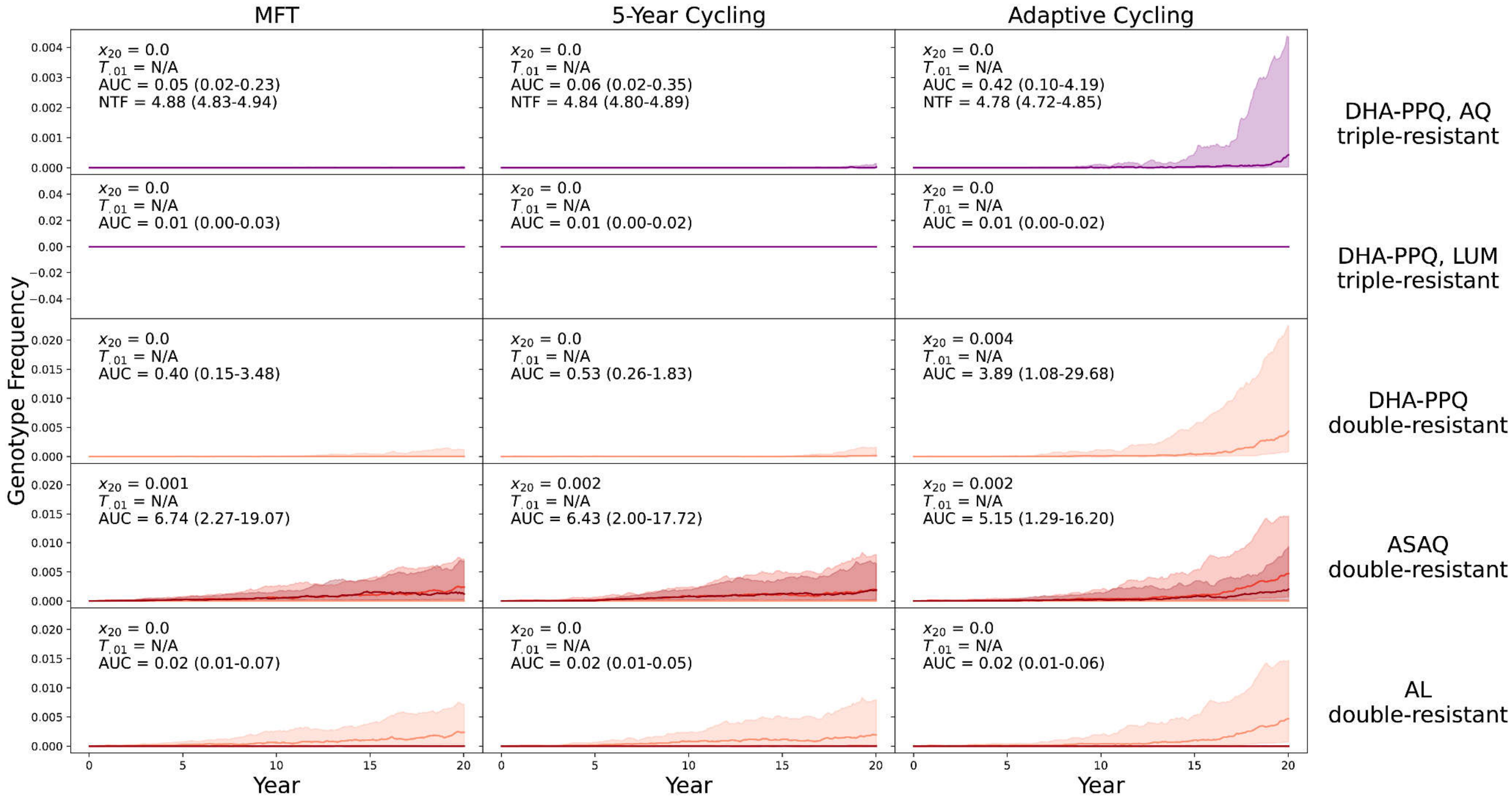

Supplementary Figure 20. PfPR$_{2\text{-}10}$ = 0.1% and treatment coverage = 20%. With Importation. Note that for the ASAQ double-resistant, MFT has a higher AUC value than either cycling strategy.

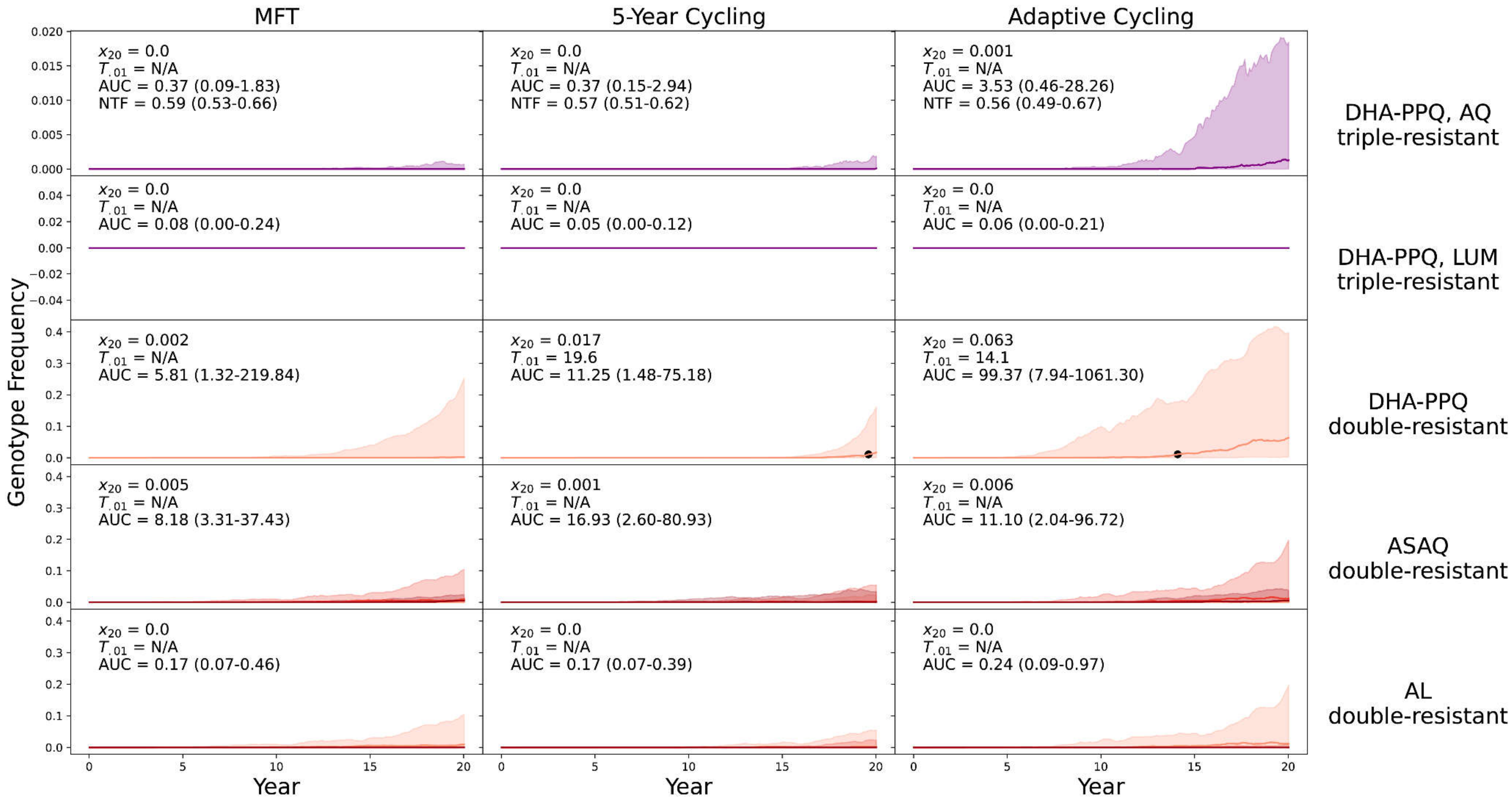

Supplementary Figure 21. PfPR$_{2\text{-}10}$ = 0.1% and treatment coverage = 40%. With Importation. Note that for the DHA-PPQ-LUM triple-resistant, MFT has a higher AUC value than either cycling strategy.

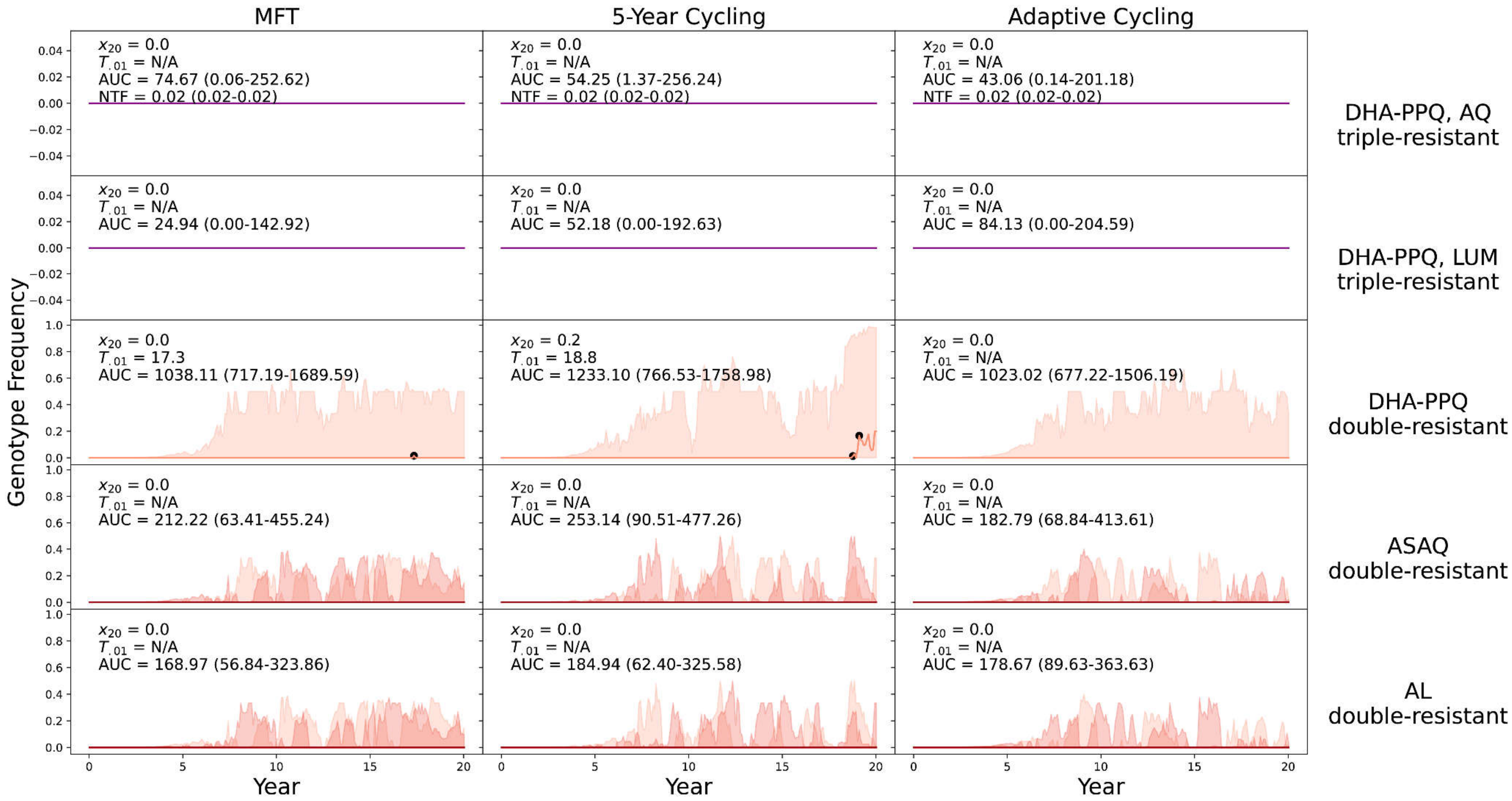

Supplementary Figure 22. PfPR$_{2\text{-}10}$ = 0.1% and treatment coverage = 60%. With Importation. Note that in 4 out of 10 comparisons MFT has a higher AUC value than cycling. This high-coverage low-prevalence scenario has the majority of its simulations runs reach extinction levels (PfPR$_{2\text{-}10}$ < 0.01% for more than 95% of runs) which corresponds to double-digit counts of parasite positive individuals. For this reason, the MDR frequencies are sometimes very high because they are being imported into a low case number environment, making the AUC values much higher than in Supplementary Figure 21 or Supplementary Figure 25.

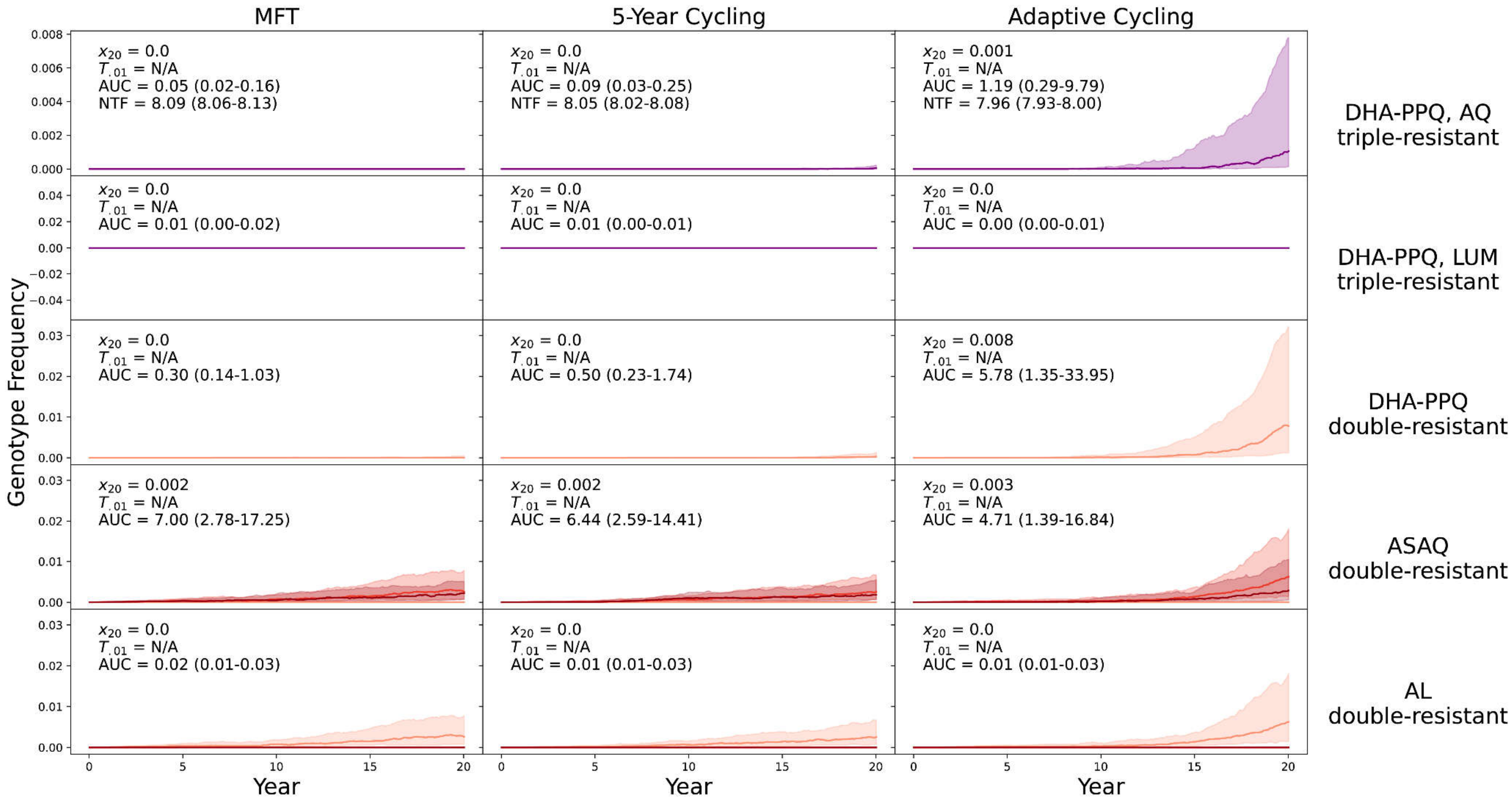

Supplementary Figure 23. $PfPR_{2\text{-}10}$ = 1% and treatment coverage = 20%. With Importation. Note that in 5 out of 10 comparisons MFT has a higher AUC value than cycling.

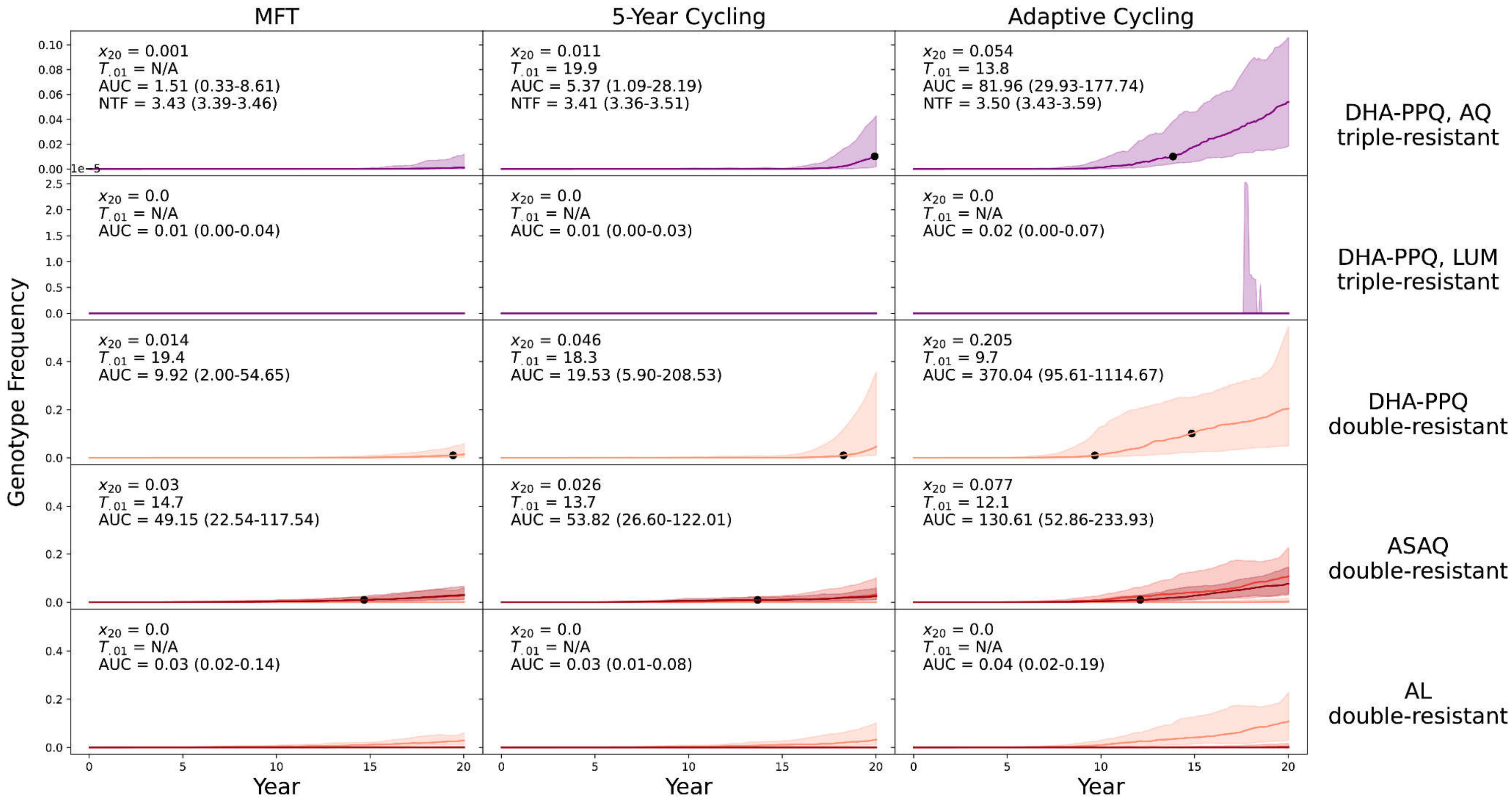

Supplementary Figure 24. $PfPR_{2-10}$ = 1% and treatment coverage = 40%. With Importation.

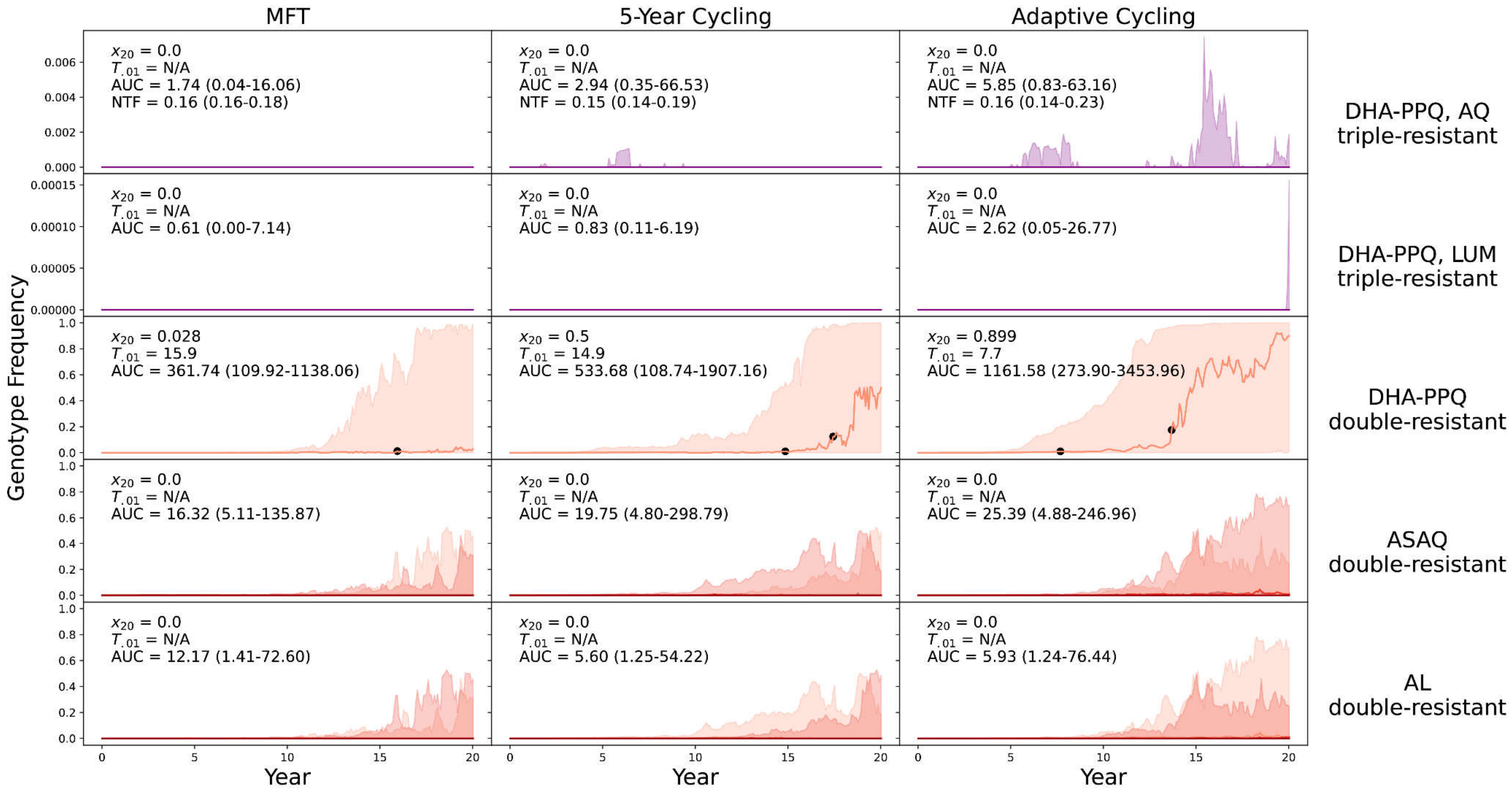

Supplementary Figure 25. $PfPR_{2\text{-}10}$ = 1% and treatment coverage = 60%. With Importation. Note that for the AL double-resistant, MFT has a higher AUC value than either cycling strategy.

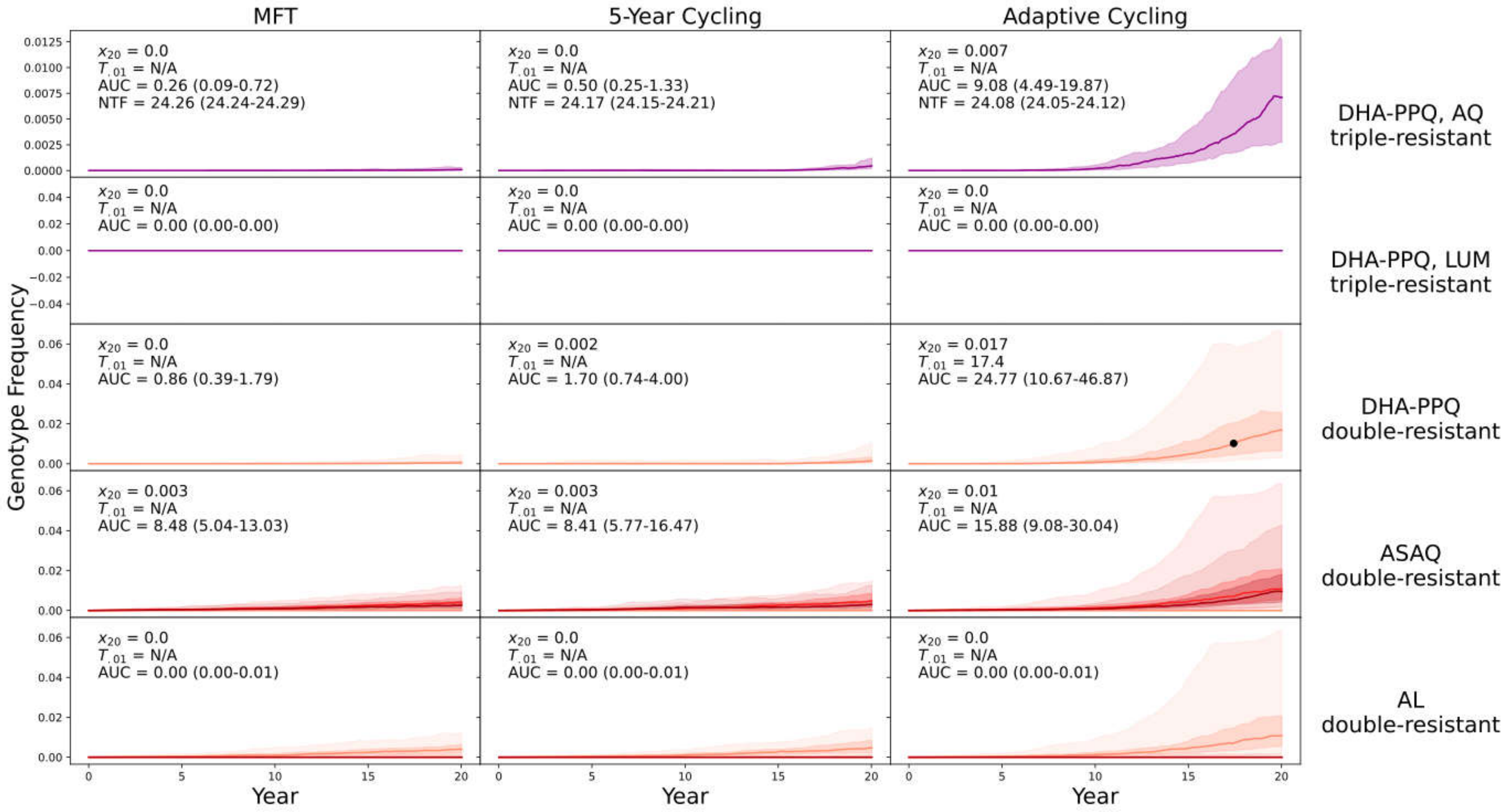

Supplementary Figure 26. PfPR$_{2\text{-}10}$ = 5% and treatment coverage = 20%. With Importation. For the ASAQ-double resistant, AUC values for MFT and 5-year cycling are nearly identical.

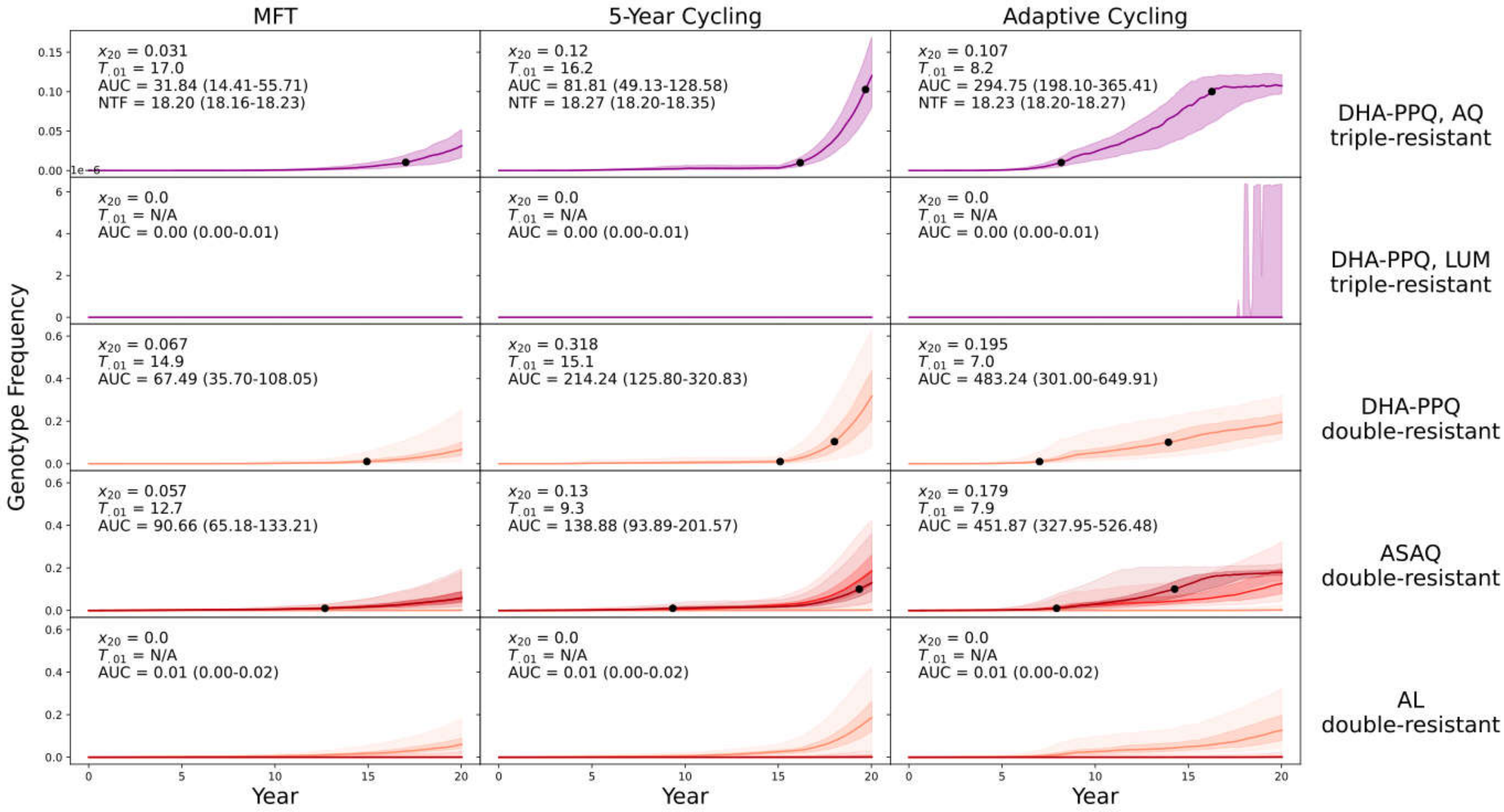

Supplementary Figure 27. PfPR$_{2\text{-}10}$ = 5% and treatment coverage = 40%. With Importation.

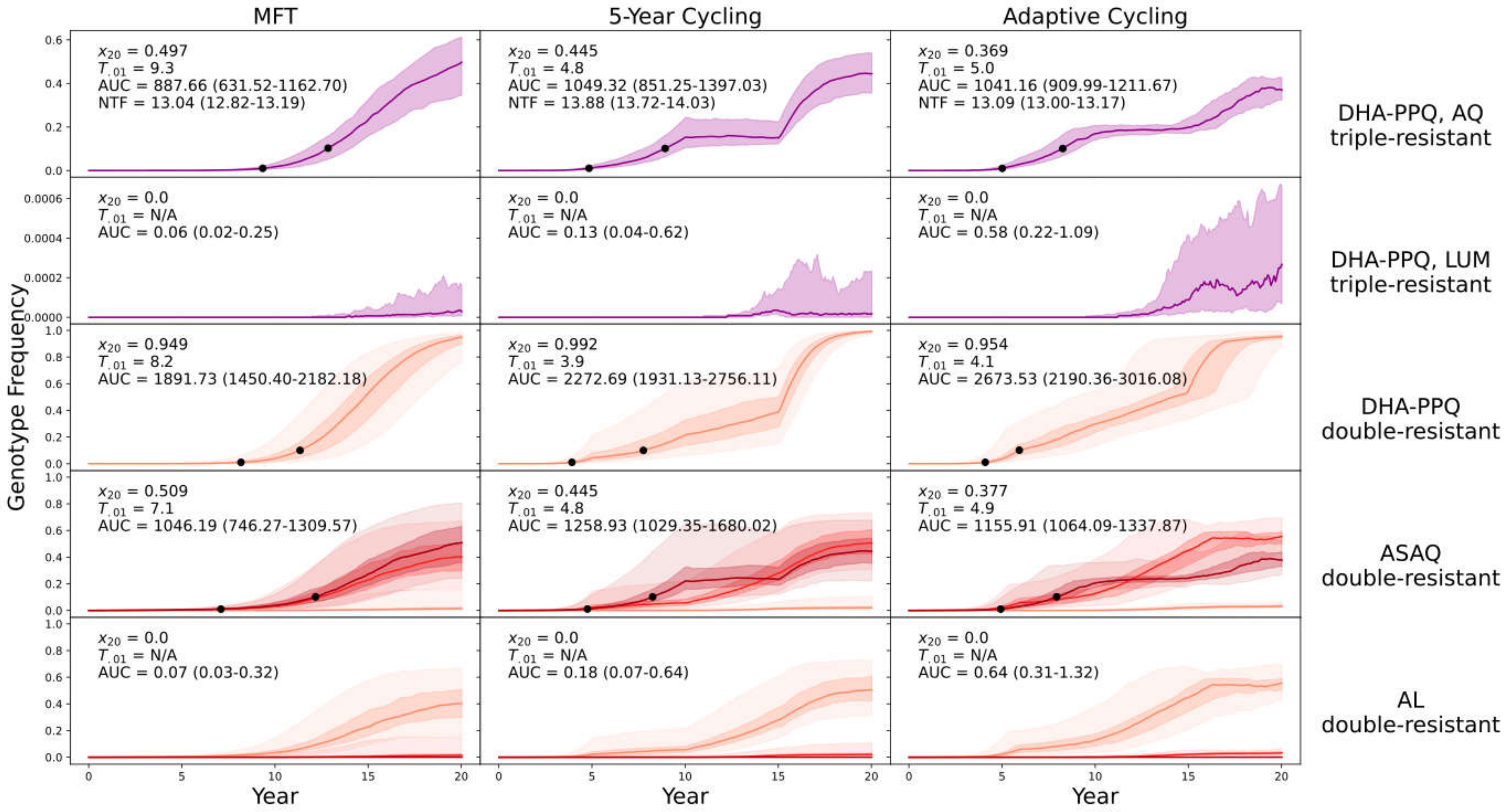

Supplementary Figure 28. PfPR$_{2\text{-}10}$ = 5% and treatment coverage = 60%. With Importation.

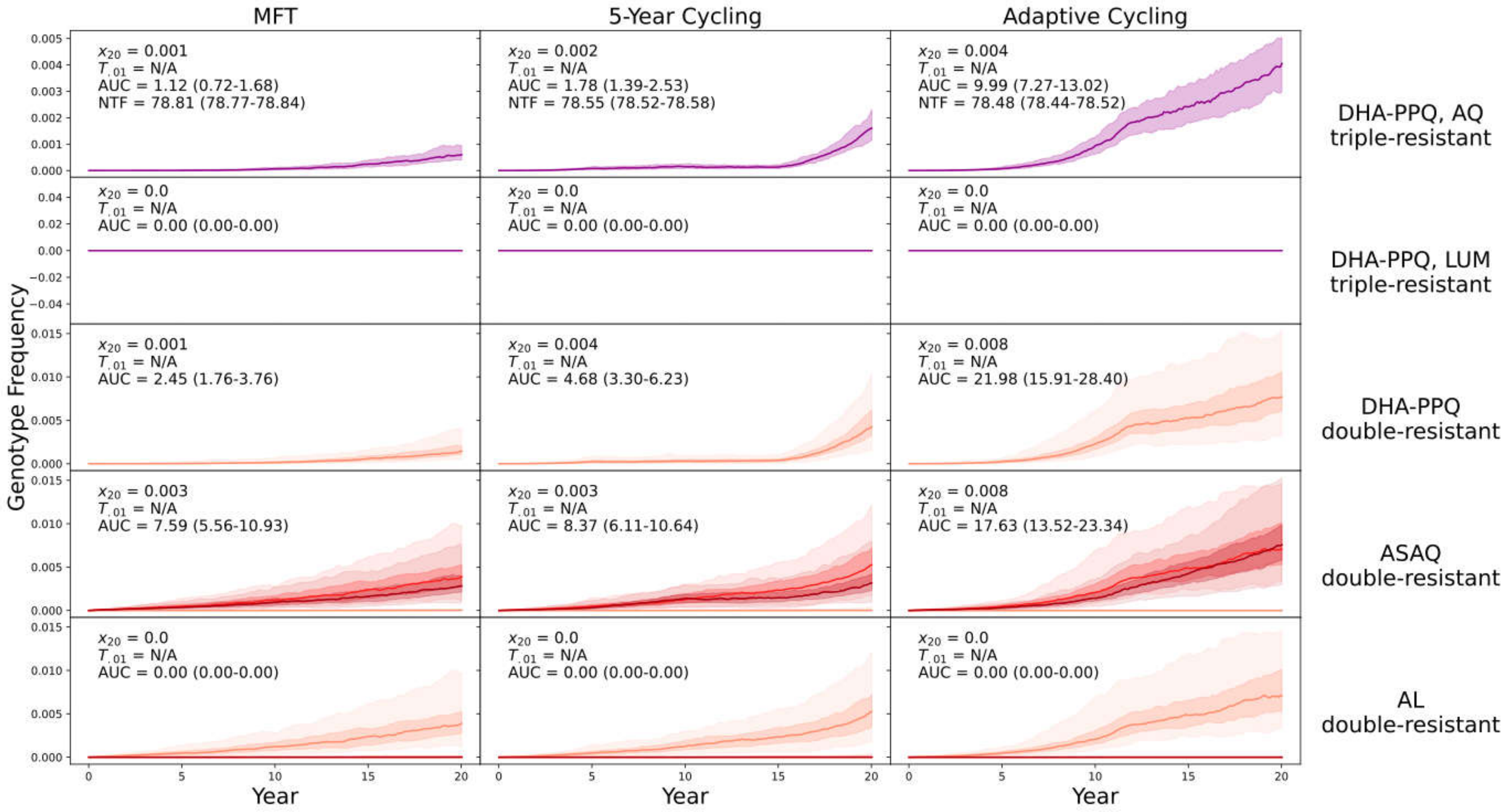

Supplementary Figure 29. PfPR$_{2\text{-}10}$ = 25% and treatment coverage = 20%. With Importation.

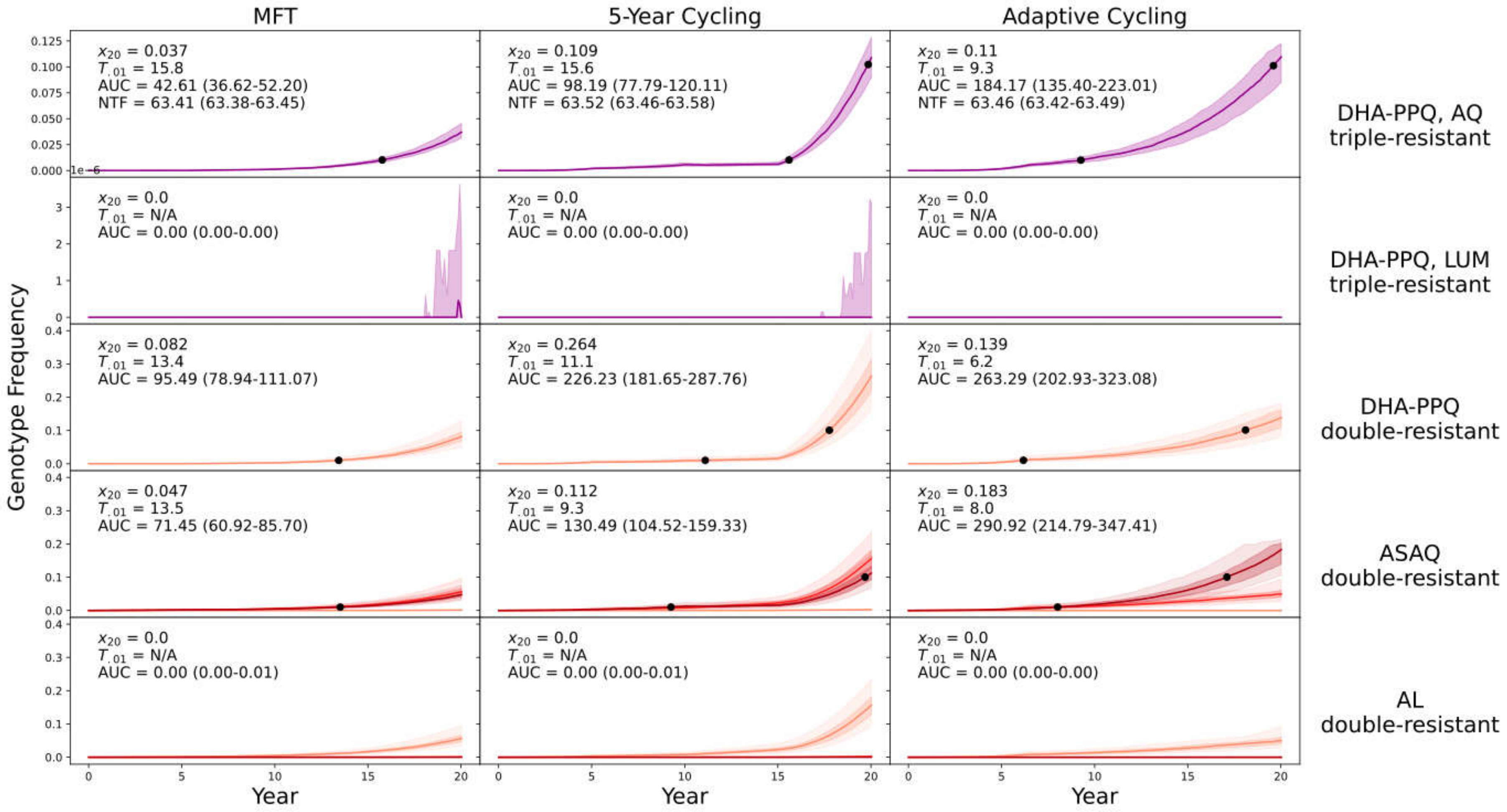

Supplementary Figure 30. $PfPR_{2\text{-}10}$ = 25% and treatment coverage = 40%. With Importation.

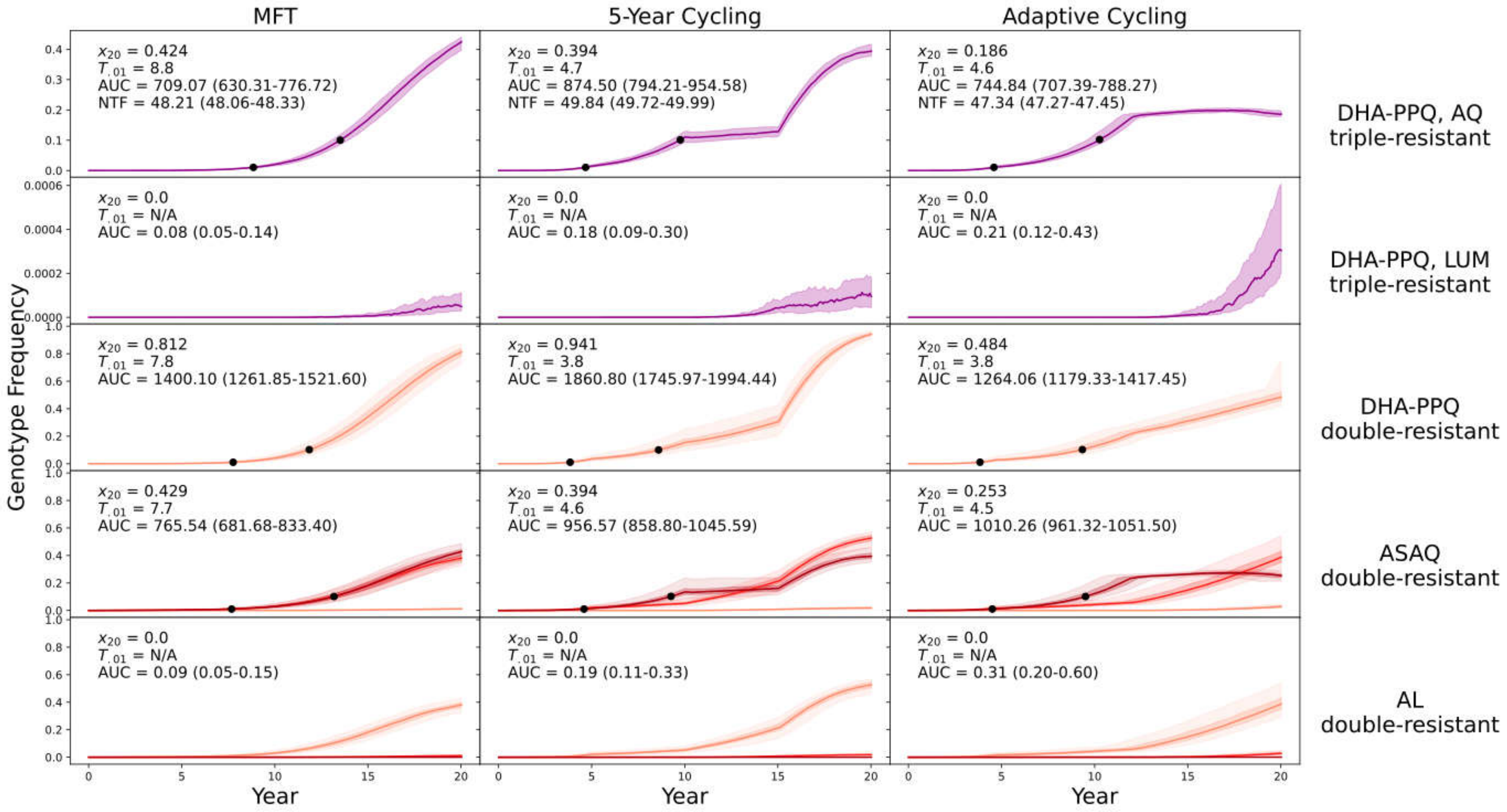

Supplementary Figure 31. $PfPR_{2-10}$ = 25% and treatment coverage = 60%. With Importation. Note that for the DHA-PPQ double-resistant, MFT has a higher AUC value than adaptive cycling.

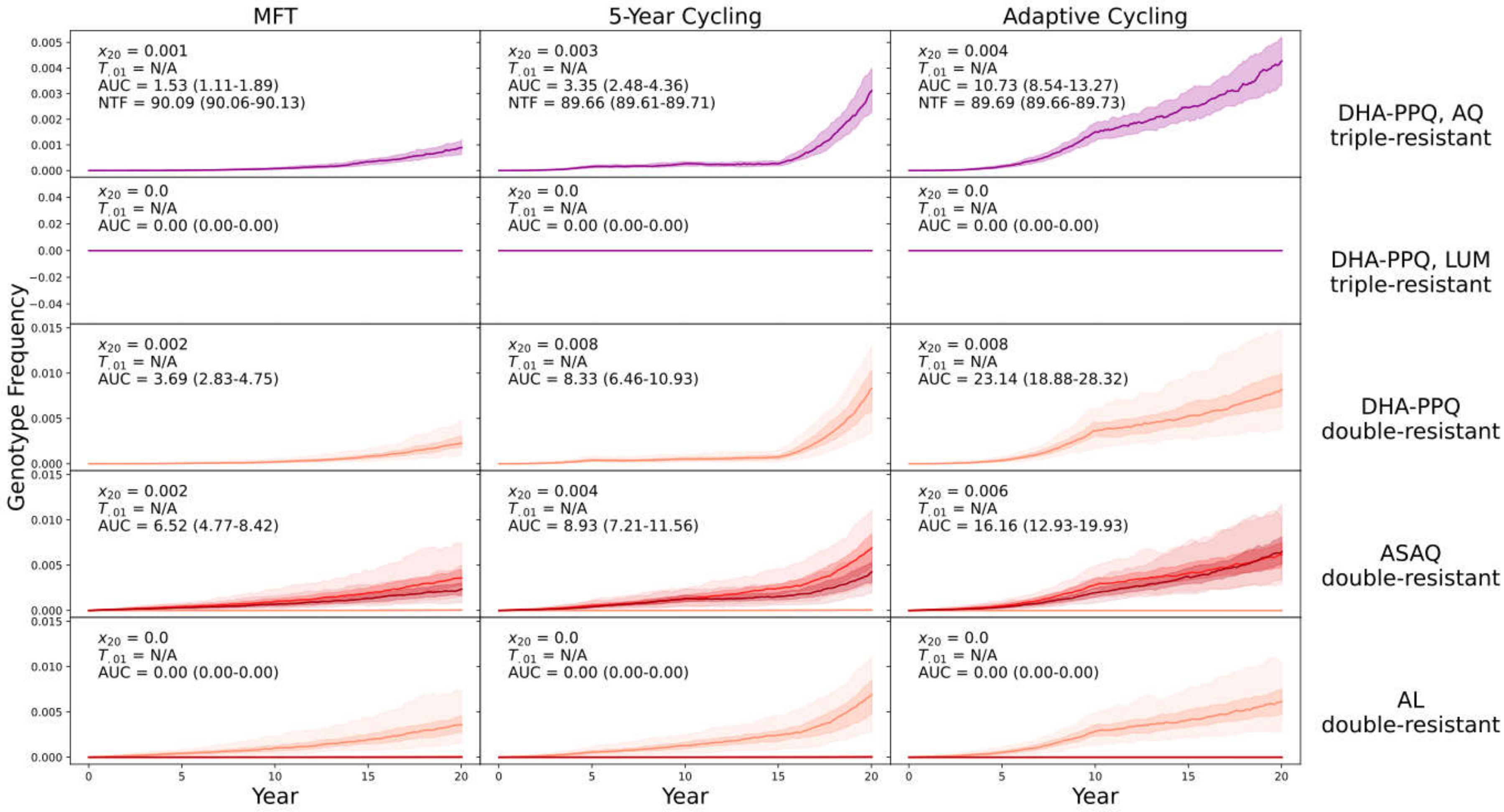

Supplementary Figure 32. PfPR$_{2\text{-}10}$ = 50% and treatment coverage = 20%. With Importation.

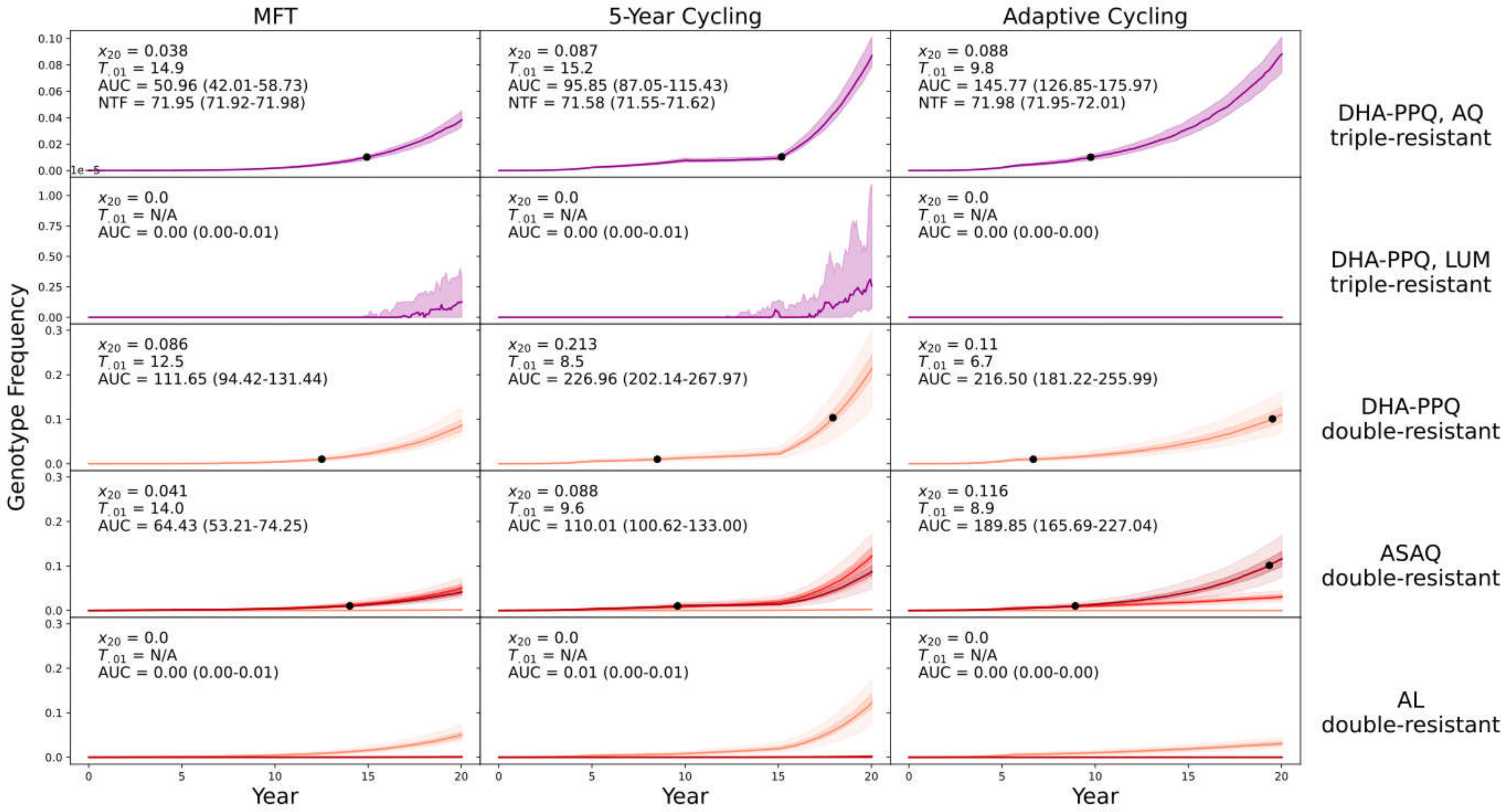

Supplementary Figure 33. PfPR$_{2\text{-}10}$ = 50% and treatment coverage = 40%. With Importation.

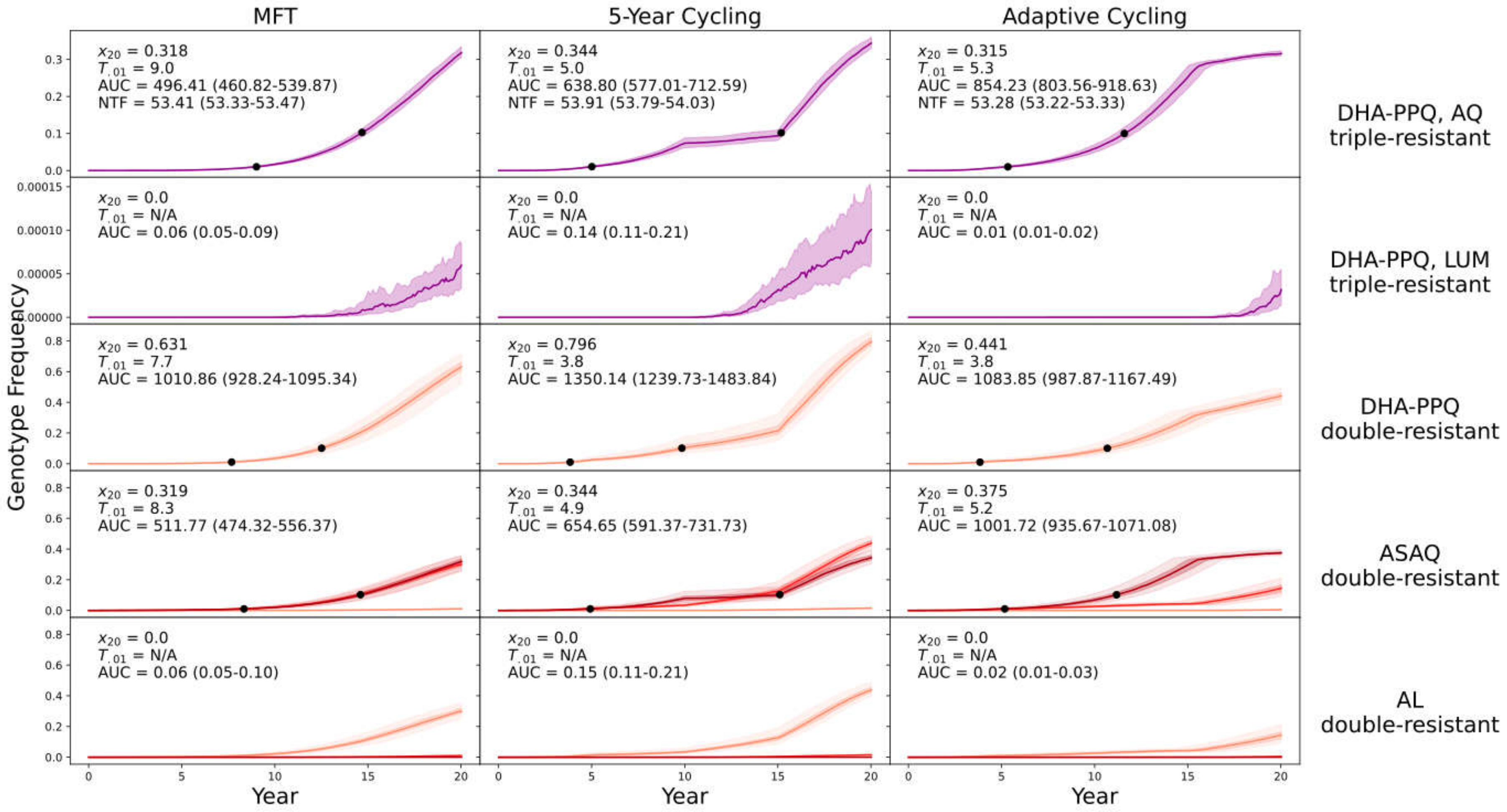

Supplementary Figure 34. PfPR$_{2\text{-}10}$ = 50% and treatment coverage = 60%. With Importation. Note that for the DHA-PPQ-LUM triple-resistant and the AL double-resistant, MFT has a higher AUC value than adaptive cycling.

Two scenarios with lower mutation rate

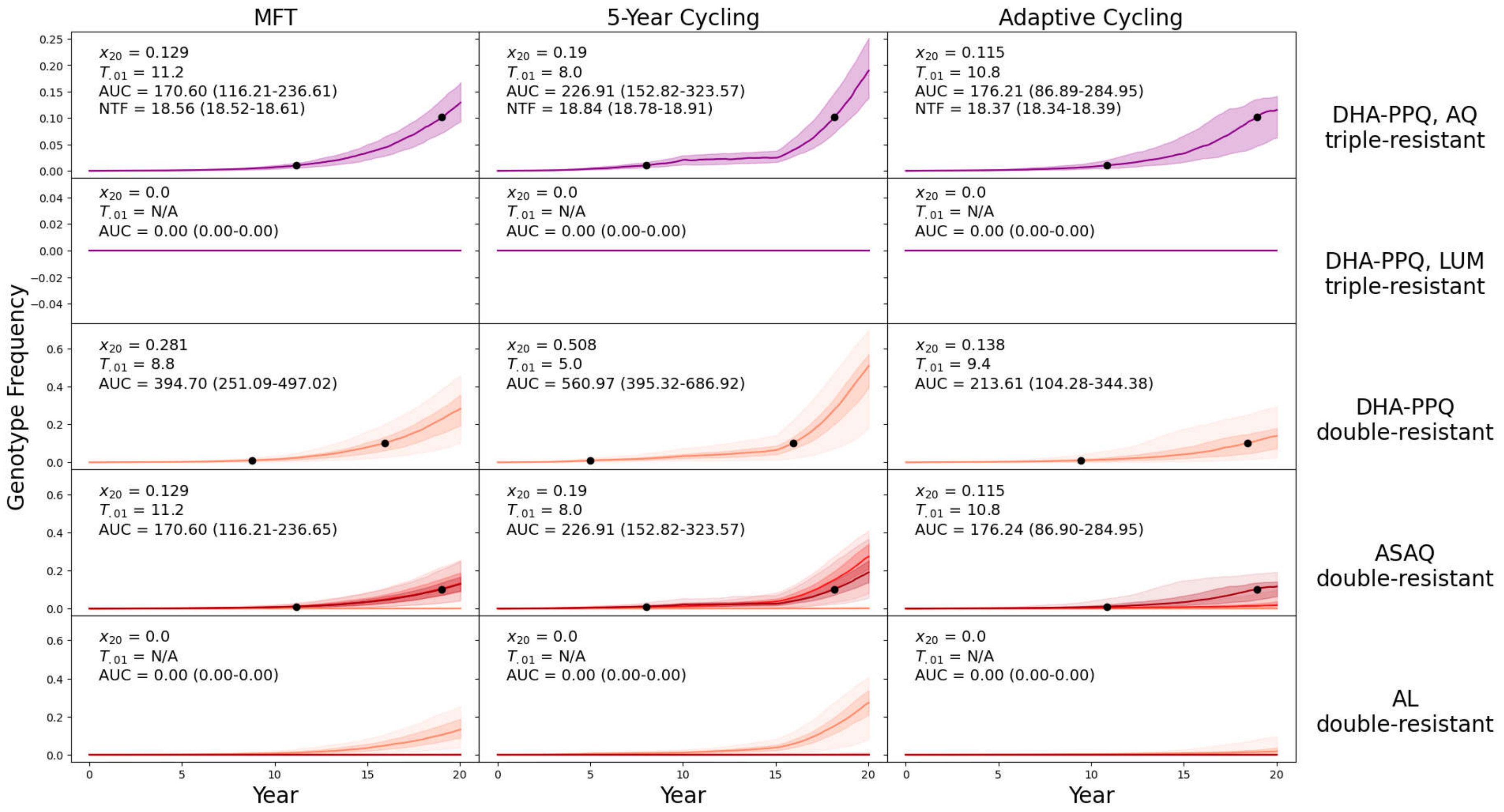

Supplementary Figure 35. $PfPR_{2\text{-}10}$ = 5% and treatment coverage = 40%. No importation. Mutation rate reduced 3-fold from 0.001983 per treated case to 0.000661 per treated case. Relationships among the strategies are similar but delay to resistance emergence is now >19 years for MFT and 5-year cycling.

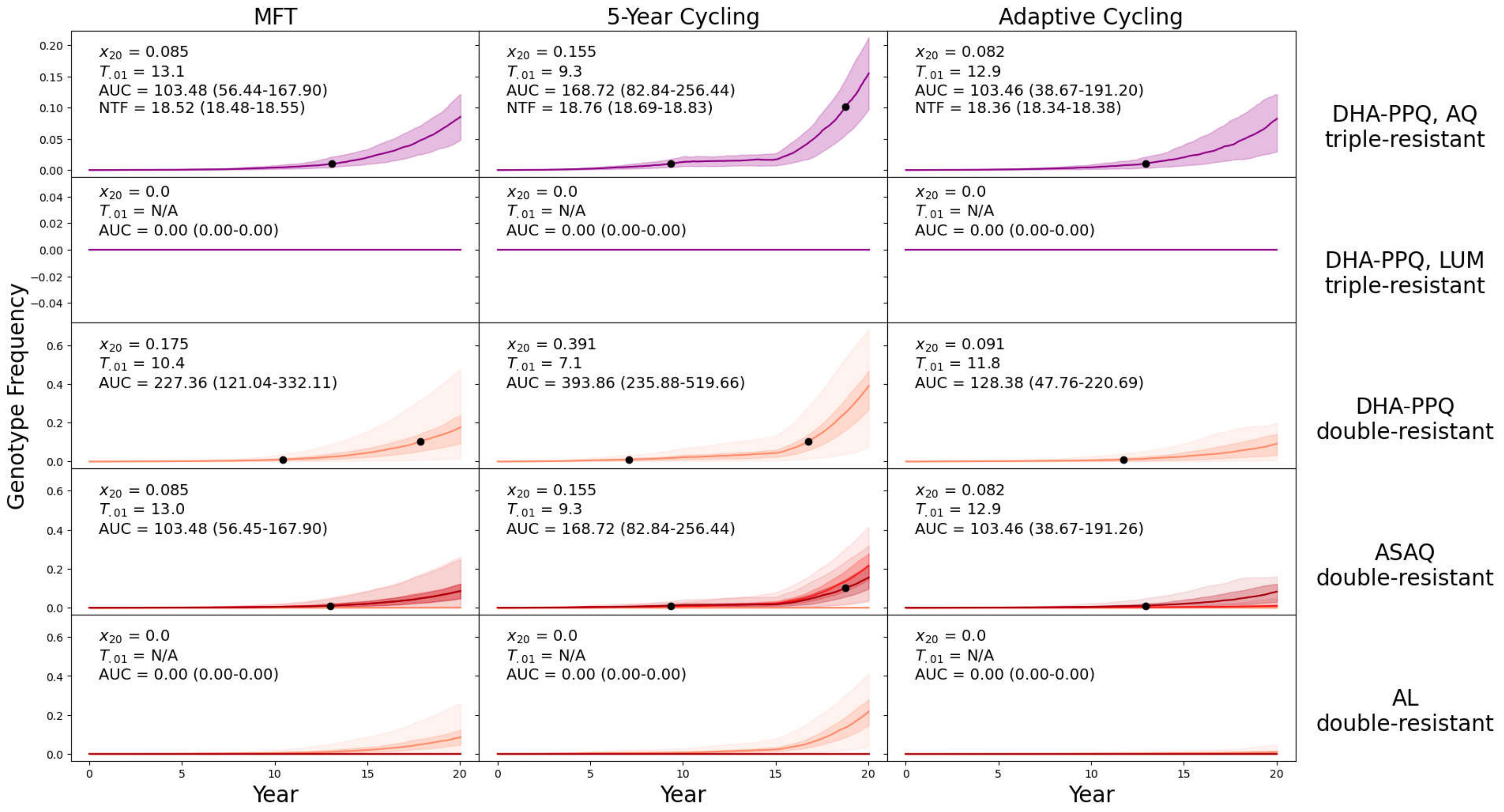

Supplementary Figure 36. PfPR$_{2\text{-}10}$ = 5% and treatment coverage = 40%. No importation. Mutation rate reduced 5-fold from 0.001983 per treated case to 0.0003966 per treated case. Relationships among the strategies are similar but delay to resistance emergence is now >20 years for MFT and 5-year cycling.

Prevalence levels for all 12 scenarios

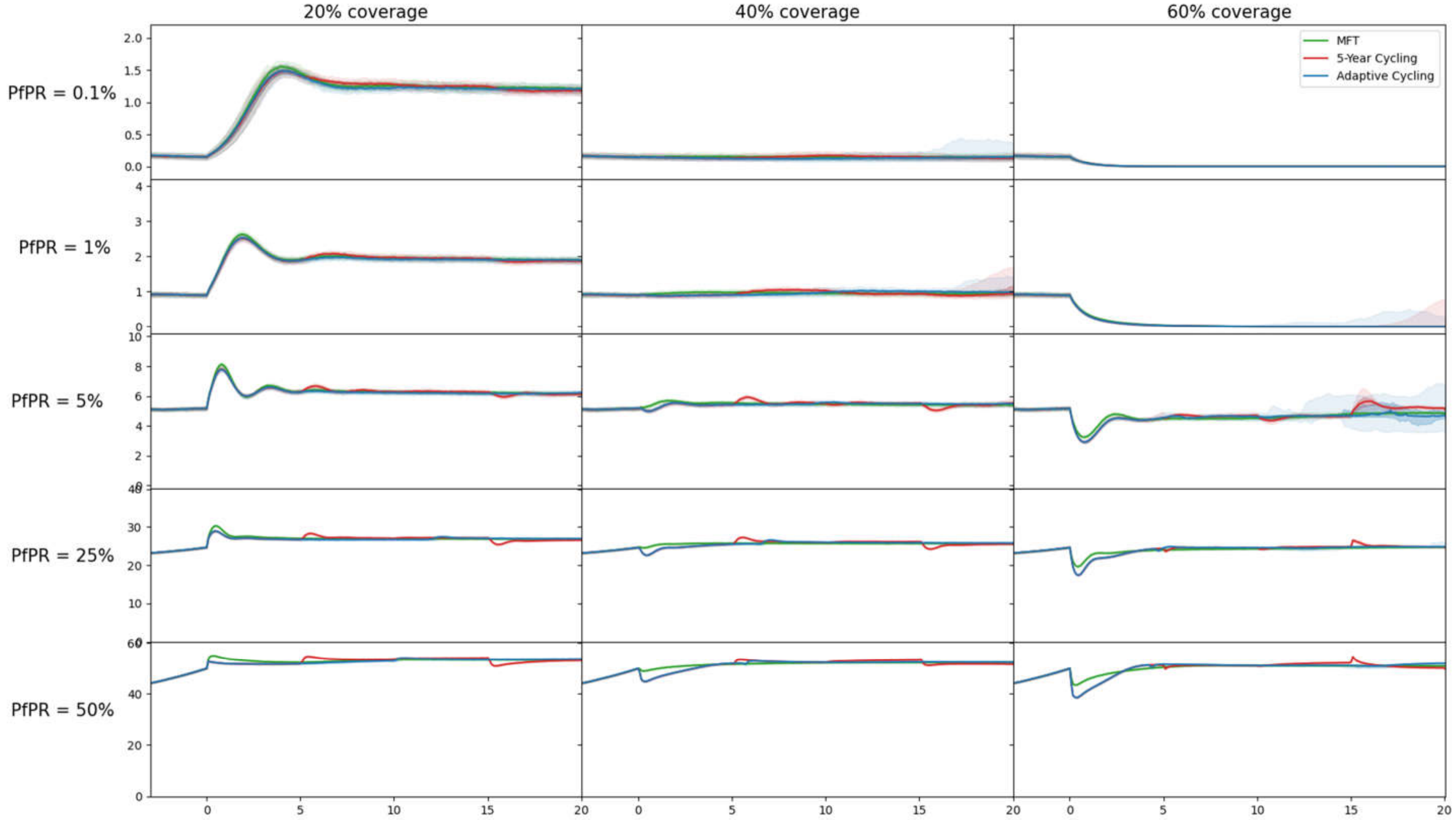

Supplementary Figure 37. Prevalence levels ($PfPR_{2\text{-}10}$) for all twelve prevalence-coverage scenarios evaluated in this analysis. Shaded area are 95% ranges. Visible shaded areas are blue (adaptive cycling) and red (5-year cycling).
