# Supplementary material for: Assessing emergence risk of double-resistant and triple-resistant genotypes of Plasmodium falciparum
Source: Nat Commun. 2024 Feb 15;15:1390. doi: 10.1038/s41467-024-45547-x (PMC10869733; doi:10.1038/s41467-024-45547-x)
Supplement: Supplementary file 3 — Reporting Summary [file 41467_2024_45547_MOESM3_ESM.pdf]

nature portfolio

Corresponding author(s): Maciej F Boni

Last updated by author(s): Jan 6, 2024

Reporting Summary

Nature Portfolio wishes to improve the reproducibility of the work that we publish. This form provides structure for consistency and transparency in reporting. For further information on Nature Portfolio policies, see our Editorial Policies and the Editorial Policy Checklist.

Statistics

For all statistical analyses, confirm that the following items are present in the figure legend, table legend, main text, or Methods section.

n/a	Confirmed	
☐	☒	The exact sample size (*n*) for each experimental group/condition, given as a discrete number and unit of measurement
☒	☐	A statement on whether measurements were taken from distinct samples or whether the same sample was measured repeatedly
☐	☒	The statistical test(s) used AND whether they are one- or two-sided<br>*Only common tests should be described solely by name; describe more complex techniques in the Methods section.*
☒	☐	A description of all covariates tested
☒	☐	A description of any assumptions or corrections, such as tests of normality and adjustment for multiple comparisons
☐	☒	A full description of the statistical parameters including central tendency (e.g. means) or other basic estimates (e.g. regression coefficient) AND variation (e.g. standard deviation) or associated estimates of uncertainty (e.g. confidence intervals)
☐	☒	For null hypothesis testing, the test statistic (e.g. *F*, *t*, *r*) with confidence intervals, effect sizes, degrees of freedom and *P* value noted<br>*Give P values as exact values whenever suitable.*
☒	☐	For Bayesian analysis, information on the choice of priors and Markov chain Monte Carlo settings
☒	☐	For hierarchical and complex designs, identification of the appropriate level for tests and full reporting of outcomes
☒	☐	Estimates of effect sizes (e.g. Cohen's *d*, Pearson's *r*), indicating how they were calculated

Our web collection on statistics for biologists contains articles on many of the points above.

Software and code

Policy information about availability of computer code

Data collection	No software was used for data collection. No new data were collected for this simulation study. Simulation outputs, or "simulated data", were used for analysis.
Data analysis	All simulation outputs, i.e. the simulated data generated for this study, are available at https://github.com/bonilab/malariaibm-generation-of-MDR-mutants. Mutation flow diagrams were generated with the Python library plotly version 5.9.0.

For manuscripts utilizing custom algorithms or software that are central to the research but not yet described in published literature, software must be made available to editors and reviewers. We strongly encourage code deposition in a community repository (e.g. GitHub). See the Nature Portfolio guidelines for submitting code & software for further information.

Data

Policy information about availability of data

All manuscripts must include a data availability statement. This statement should provide the following information, where applicable:

- Accession codes, unique identifiers, or web links for publicly available datasets
- A description of any restrictions on data availability
- For clinical datasets or third party data, please ensure that the statement adheres to our policy

All simulation outputs, i.e. the simulated data generated for this study, are available at https://github.com/bonilab/malariaibm-generation-of-MDR-mutants

Research involving human participants, their data, or biological material

Policy information about studies with human participants or human data. See also policy information about sex, gender (identity/presentation), and sexual orientation and race, ethnicity and racism.

Reporting on sex and gender	No human subjects involved in this study
Reporting on race, ethnicity, or other socially relevant groupings	No human subjects involved in this study
Population characteristics	No human subjects involved in this study
Recruitment	No human subjects involved in this study
Ethics oversight	No human subjects involved in this study

Note that full information on the approval of the study protocol must also be provided in the manuscript.

Field-specific reporting

Please select the one below that is the best fit for your research. If you are not sure, read the appropriate sections before making your selection.

☐ Life sciences ☐ Behavioural & social sciences ☒ Ecological, evolutionary & environmental sciences

For a reference copy of the document with all sections, see nature.com/documents/nr-reporting-summary-flat.pdf

Ecological, evolutionary & environmental sciences study design

All studies must disclose on these points even when the disclosure is negative.

Study description	A simulation study determining whether double-resistance or triple-resistance emerge earlier/later under different drug distribution strategies. Two major "treatment factors" are malaria prevalence and drug coverage.
Research sample	100 simulations each for 30 major epidemiological scenarios, with three drug-distribution strategies evaluated for each scenario.
Sampling strategy	Sample sizes of 100 simulations per scenario were chosen for feasibility and timely completion of research.
Data collection	No new data collected for this study.
Timing and spatial scale	Simulation evaluations are over a 20-year period. The population is viewed as one well-mixed population (with no special spatial structure).
Data exclusions	No data (i.e. simulation outputs) were excluded from the study.
Reproducibility	Fully reproducible with source code at https://github.com/bonilab/malariaibm-generation-of-MDR-mutants
Randomization	It is not necessary to randomize simulation studies. Computer code runs deterministically with no possibility of a placebo effect.
Blinding	This was a simulation study. The investigators are not blinded to the analysis but the outcomes are fully reproducible.

Did the study involve field work? ☐ Yes ☒ No

Reporting for specific materials, systems and methods

We require information from authors about some types of materials, experimental systems and methods used in many studies. Here, indicate whether each material, system or method listed is relevant to your study. If you are not sure if a list item applies to your research, read the appropriate section before selecting a response.

Materials & experimental systems

n/a	Involved in the study
☒	☐ Antibodies
☒	☐ Eukaryotic cell lines
☒	☐ Palaeontology and archaeology
☒	☐ Animals and other organisms
☒	☐ Clinical data
☒	☐ Dual use research of concern
☒	☐ Plants

Methods

n/a	Involved in the study
☒	☐ ChIP-seq
☒	☐ Flow cytometry
☒	☐ MRI-based neuroimaging

Plants

Seed stocks	N/A
Novel plant genotypes	N/A
Authentication	N/A
